# Synthesis and Characterisation of Fluorescent Novel Pt(II) Cyclometallated Complexes with Anticancer Activity

**DOI:** 10.3390/ijms24098049

**Published:** 2023-04-28

**Authors:** Brondwyn S. McGhie, Jennette Sakoff, Jayne Gilbert, Christopher P. Gordon, Janice R. Aldrich-Wright

**Affiliations:** 1 School of Science, Nanoscale Organisation and Dynamics Group, Western Sydney University, Locked Bag 1797 Penrith South DC, Penrith, NSW 2751, Australia; 2 Calvary Mater Newcastle, Waratah, Newcastle, NSW 2298, Australia

**Keywords:** cyclometallated, anticancer, platinum

## Abstract

Cancer poses a significant threat to global health and new treatments are required to improve the prognosis for patients. Previously, unconventional platinum complexes designed to incorporate polypyridyl ligands paired with diaminocyclohexane have demonstrated anticancer activity in KRAS mutated cells, previously thought to be undruggable and have cytotoxicity values up to 100 times better than cisplatin. In this work, these complexes were used as inspiration to design six novel cyclometallated examples, whose fluorescence could be exploited to better understand the mechanism of action of these kinds of platinum drugs. The cytotoxicity results revealed that these cyclometallated complexes (CMCs) have significantly different activity compared to the complexes that inspired them; they are as cytotoxic as cisplatin and have much higher selectivity indices in breast cancer cell lines (MCF10A/MCF-7). Complexes **1b**, **2a**, and **3b** all had very high selectivity indexes compared to previous Pt(II) complexes. This prompted further investigation into their DNA binding properties, which revealed that they had good affinity to ctDNA, especially CMCs **1a** and **3b**. Their inherent fluorescence was successfully utilised in the calculation of their DNA binding affinity and could be useful in future work.

## 1. Introduction

In over 120 countries, cancer is ranked within the top three causes for premature death, and, in 2020 alone, cancer was responsible for close to 10 million deaths worldwide [1,2,3]. These figures are expected to worsen, with cancer expected to become the number one cause of premature death worldwide by the end of the century, and as such there is a clear and increasing need for more efficient cancer treatments. Although this is a centuries old problem, with more treatment options released almost yearly, the current prognosis for cancer patients is less than ideal, particularly for those in the later stages of the disease [4,5,6]. Platinum(II)-based chemotherapies are still among the most commonly used chemotherapeutics, with cisplatin alone included in 40% of cancer treatments, and improving these complexes is part of a broad area of cancer research, working to reduce resistance and improve targeting [7,8,9,10].

**Figure 1:**
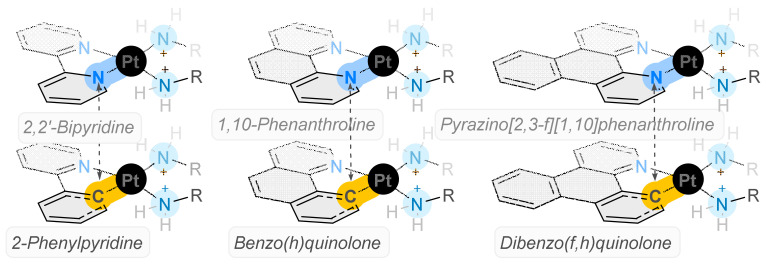
Polypyridal ligands (P_L_) previously incorporated in [Pt(P_L_)(A_L_)]^+2^ complexes [11,12,13] and the comparable cyclometallated counterpart (C_L_).

To this end, several Pt(II) complexes have been identified as potential next-generation platinum-based chemotherapeutic agents. These complexes have the basic structure [Pt(P_L_)(A_L_)]^+2^, featuring a polypyridyl ligand (P_L_)and an ancillary ligand (A_L_)—usually *SS* or *RR* diamino cyclohexane—and exhibit a cytotoxicity over 100-fold greater than cisplatin [11,12,13,14]. These complexes possess a unique mechanism of action which diminishes the likelihood of cross-resistance with cisplatin. Nevertheless, in response to the near-inevitable emergence of resistance against cisplatin-based complexes, it is essential to continuously develop diverse sets of complexes to counteract resistance. The current study presents the synthesis of novel cyclometallated complexes (CMCs), where the polyaromatic ligand has been replaced with an analogous cyclometallated ligand (Figure 1). Although these complexes share certain characteristics, they exhibit unique electrochemical properties, with the carbon–platinum bond demonstrating notably higher strength compared to the nitrogen–nitrogen bond. These complexes represent a distinct addition to the existing literature, as previous research on cyclometallated complexes has primarily focused on applications such as catalysis, organic light-emitting diodes (OLEDs), electrical conductors, optoelectronic devices, and molecular materials, with only a handful of examples pertaining to medicinal applications [15,16,17,18,19,20,21,22,23,24,25].

**Figure 2:**
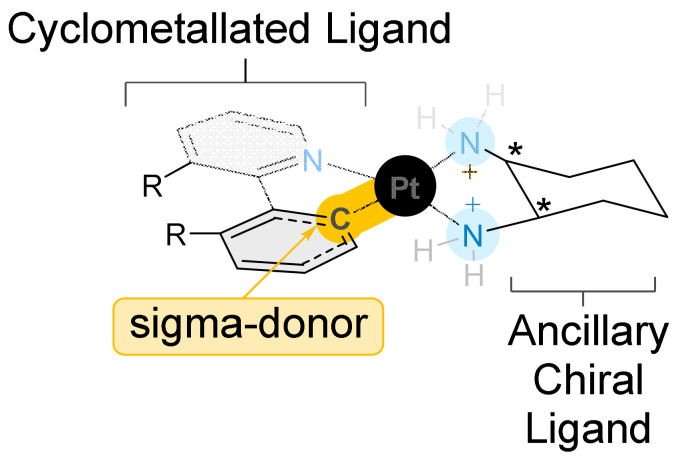
General structure of CMCs where * indicates stereocenter.

On account of the purported relatively subtle conformational variation imparted, it was hypothesised that the planar CMC’s unique composition will preserve the cytotoxicity expressed by the reported [Pt(P_L_)(A_L_)]^+2^ complexes [11,12,13], while the alteration of the structure, electrochemistry, and strength of the bond between the polyaromatic ligand and the Pt centre will alter the mechanism of action significantly enough that they will not be cross-resistant. The inherent fluorescence gained by the cyclometallation introduces a new advantage that may be used to better explore their mechanism of action, and potentially in the future to reveal the intercellular target/s, which would facilitate the elucidation of the means of action [26,27,28,29,30]. The coordination of platinum to the C1 of the cyclometallated ligand to form an organo-metallic interaction results in the carbon becoming a strong σ-donor, which creates a strong ligand field with the strong π-accepting polypyridyl group. The increased energy gap between the occupied and unoccupied orbitals results in fluorescence (Figure 2).

**Figure 3:**
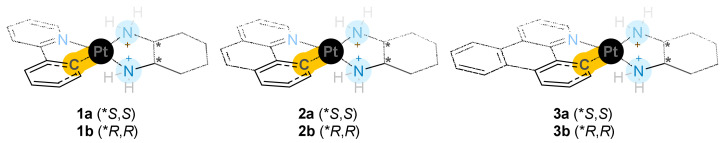
Structure of polyaromatic cyclometallated complexes **1**–**3**, * indicates stereocenter, either *SS* or *RR* referred to as a or b, respectively.

Herein, we report the synthesis, characterisation, and preliminary biophysical properties of six resolved chiral square planar cyclometallated complexes that incorporate the structure of highly cytotoxic previously synthesised platinum(II) complexes and the added interest of a cyclometallated ligand, which brings fluorescence and varied electrochemistry to the complex (Figure 3). The resulting complexes are expected to exhibit good emissive properties and very promising GI_50_ values. In this work, the interaction with calf thymus DNA (ctDNA) was ascertained using a combination of fluorescence, UV, and CD experiments, while the inherent fluorescence was determined by emission quantum yield.

## 2. Results and Discussion

### 2.1. Synthesis and Characterisation

The synthetic strategy for these complexes was derived from the aforementioned [Pt(P_L_)(A_L_)]^+2^ type complexes. However, instead of initially synthesising [Pt(A_L_)Cl_2_]^+2^ [31], the cyclometallated ligand was reacted with the platinum first before refluxing with the cyclometallated ligand. The original methodology resulted in far lower yields due to the formation of a solid black precipitate, which was avoided using the novel method described above. Impurities could also be reduced by sonicating the reaction mixture after 4 h to reincorporate any residue that was stuck to the glass and then returning it to the reflux apparatus for the remaining time.

Each complex was characterised using a combination of NMR spectroscopy, HPLC, circular dichroism, UV spectroscopy, and ESI-MS. The NMR characterization of the complex was achieved using a combination of ^1^H proton NMR spectra and ^1^H-^195^Pt heteronuclear multiple quantum correlation (HMQC) spectra. The NMR spectra produced peaks consistent with those seen in the literature for similar compounds with little to no impurities detected, as confirmed with HPLC [11,12,13,28,29,32]. The CD spectra confirmed that the chirality of the starting materials was retained during synthesis. Additionally, both the UV and CD spectra revealed dramatic differences to similar, previously published, Pt(II) complexes N^N complexes [11,12,13]. The predicted mass peak was identified using ESI-MS, which, combined with the other characterisation, confirms the successful synthesis as summarised in Table 1.

UV absorption was used to determine the extinction coefficient of the complexes in water. The UV spectra of the cyclometallated ligands were also collected and compared to those of the six complexes. Unsurprisingly, the three ligands contribute to significantly different bands in the spectra, and these defining peaks were evident in the spectra of the resulting complexes. The spectra of the 2-phenylpyridine ligand had a strong band at ~250 nm and this was evident the spectra of both **1a** and **1b**. A second band at ~280 nm was observed as a shoulder in the spectra of the complexes and was blue-shifted. A similar pattern was observed for benzo(h)quinoline and dibenzo(f,h)quinoline, where the defined ligand bands became less defined and slightly blue-shifted upon being coordinated in **2a/b** and **3a/b**, respectively.

Fluorescence is increasingly becoming a point of interest in medicinal chemistry, with molecules’ photodynamic properties being used for targeting, tracking, photoactivation, theragnostics, and ROS generation [30,33,34,35]. The fluorescence excitation and emission maxima (Em_max_ and Ex_max_) were determined for each complex (Table 2). The stereochemistry of the dichlorocyclohexane appears to have a significant impact on fluorescence. The ‘**a**’ complexes with *SS* configuration had lower Ex_max_ wavelengths (between 262 and 287 nm) than their ‘**b**’ *RR* counterparts. Interestingly, the stereochemistry impacted the Em_max_ only for complexes **2a/b**. Radiative quantum yield (Φ_F_) similarly showed the impact of the *SS* vs. *RR* conformation; in this case, the **2a** and **3a** complexes tended to have lower Φ_F_ values than the ‘**b**’ complexes. The Φ_F_ values increased with the size of the ligand, except for **3a**. Alternatively, the difference in fluorescence could be explained by the electron availability of the ligand. Unfortunately, the emission and excitation maxima are in the same range as autofluorescence, and are not within the optic or photodynamic window that would enable the CMCs fluorescence to be utilised in tissue [30].

### 2.2. Biophysical Characterisation

To investigate the potential mechanisms of action of complexes **1**–**3**, fluorescent intercalator displacement (FID) experiments and DNA binding experiments utilizing the innate fluorescence were undertaken. For FID experiments, the concentration of a fluorescent intercalator, in this case ethidium bromide (EtBr), that is displaced by the complex under investigation is determined. In a solution of calf-thymus DNA (ctDNA) where is saturated with EtBr, the change in fluorescence is monitored as the complex is titrated into this solution. Once the decrease in fluorescence has stopped, the concentration of the complex allows us to calculate the stoichiometric point of binding, the change in fluorescence at the point of binding (ΔFsat), the binding coefficient (Ka), bimolecular quenching constant (Kq), Stern–Volmer quenching constant (Ksv), the binding constant for fluorescence (K_F_), and the molar equivalents of CMC (n) at the point of intersection is the experimental stoichiometry of binding.

(1) $${\left[ctDNA\right]}_{T}\left[n-\frac{∆F_{x}}{∆F_{sat}}\right]=[CMC]$$

(2) $$\left(\frac{∆F_{x}}{∆F_{sat}}\right)\frac{1}{n}=\text{fraction of ctDNA}-\text{CMC complex}$$

(3) $$1-\left(\frac{∆F_{x}}{∆F_{sat}}\right)\frac{1}{n}=\text{fraction free CMC}$$

The fluorescence at 601 nm for each titration was plotted, and the K_F_ and n could be calculated from the intercept and slope, respectively.

(4) $$\frac{F_{0}}{F}=1+K_{q}\tau_{0}\left[CMC\right]=1+K_{SV}[CMC]$$

where F_0_ is the fluorescence of the binding site in the absence of quencher (CMC), F is the fluorescence of the site containing the CMC, and t_0_ is the lifetime of the chromophore in the absence of the quencher (Ɛ_476_ = 5680 for ethidium-bound DNA).

A plot of F_0_/F against [CMC] using experimental values allows for the determination of K_q_ and K_SV_ from the slope.

(5) $${log}_{10}\left(\frac{F_{0}-F}{F}\right)=n{log}_{10}\left[CMC\right]+{log}_{10}K_{F}$$

where n is the number of ethidium ligands that are displaced per CMC. It is important to note that this expression is a simplification of the true binding interaction, as the effect of EtBr on the binding equilibrium is ignored; however, the results obtained were comparable between complexes in this study. A plot of log_10_[(F_0_ − F)/F)] against log_10_[CMC] is used to determine K_F_ and n from the intercept and slope, respectively. Experiments were performed in triplicate for each CMC. Results summarized in Table 3.

ctDNA experiments utilising the inherent fluorescence of the complexes were completed in addition to the FID experiments. The values of Ka of compounds can be determined based on the Benesi–Hildebrand equation (Equation (6)) as follows:

(6) $$\frac{1}{F-F_{0}}=\frac{1}{K_{a}\left(F_{I}-F\right){\left[CMC\right]}^{n}}+\frac{1}{F_{I}-F}$$

where F_I_ are the fluorescence intensities of the compound ctDNA complexes, Ka is the binding constant, and n is the binding ratio. The values of Ka and n for the compounds are listed in Table 4, where R^2^ is the linear correlation coefficient for Ka. From the results, the Ka value of the compounds is in a range of 11,086–36,842 molL^−1^, which shows that the binding affinity of the complexes and ctDNA is relatively strong.

The two methods of finding the binding coefficient produced different numbers; however, when plotted together, they correlate with an R^2^ value of 0.96 (Supplementary Figure S58). The differences can therefore be explained by the presence of the EtBr, whereby the CMC is competing with the strength of the binding constant of the EtBr. However, EtBr binds in several locations [36] with increasing binding affinity and an average of these is used to calculate the binding affinity of the CMC, which explains why direct measurement of the CMCs fluorescence may result in slightly different and presumably more accurate results. The FID experiments were still valuable in this case in order to allow equal comparison with other non-cyclometallated Pt(II) complexes and to validate the results of the inherent fluorescence experiment.

### 2.3. CD ctDNA Experiments

**Figure 4:**
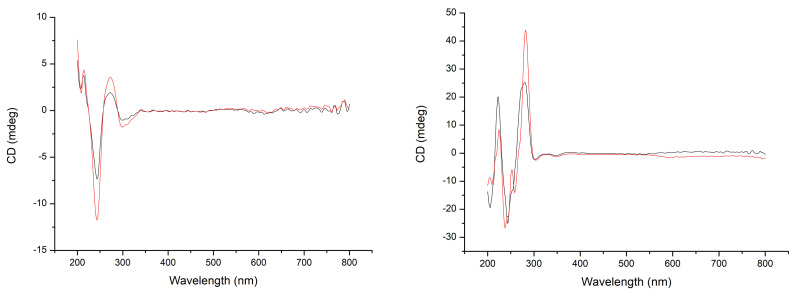
CD spectra of ctDNA at one (black) and two (red) equivalents for CMC **2b** (**right**) and **3b** (**left**).

Circular dichroism experiments were undertaken where the CMC was exposed to one and two equivalents of ctDNA to confirm binding. Figure 4 shows that there was an increase in signal when DNA equivalents were increased, but the peaks did not shift up- or downfield. These experiments confirmed the results of the DNA binding experiments above, in that these unique CMCs are good DNA binders, suggesting that this may contribute to their mechanism of action.

### 2.4. Cytotoxicity

**Figure 5:**
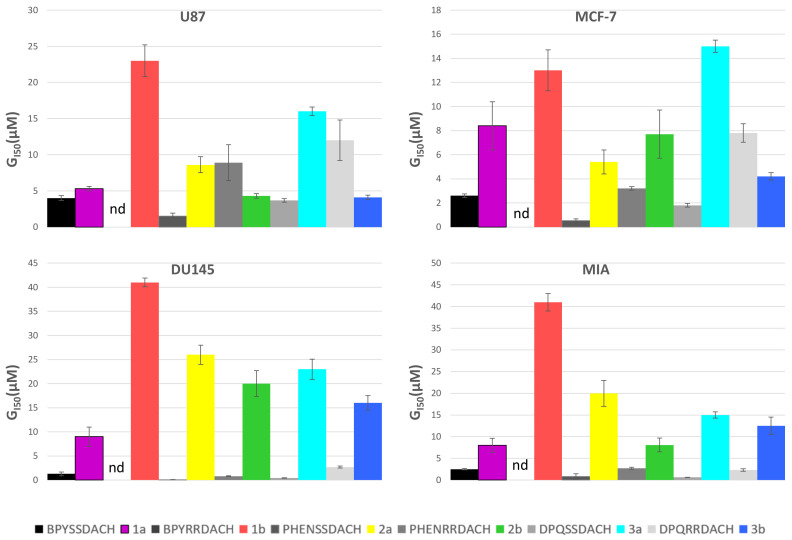
GI_50_ data for complexes **1**–**3** compared to their inspiration complexes pictured in Figure 1 where the CMCs are in colour and the inspiration complexes are in greyscale. nd = no data available.

Cytotoxicity experiments we performed against a diverse panel of cell lines including one non-cancer cell line MCF10A. The resulting GI_50_ values are comparable to those of cisplatin but are significantly higher compared with 56MESS, the complex on which these CMCs were based (Table 5). Although less cytotoxic, the CMCs have a much higher selectivity index, meaning that they are more effective at killing cancer cells than normal cells in breast cancer cell lines. For CMCs **1b**, **2a**, and **3b**, the selectivity index is over two, indicating that in breast cancer cell lines MCF-7 and MCF10A these complexes are selective towards the cancer cell line. When plotted compared to the polypyridyl DACH complexes with similar ligands as described in Figure 1, a similar trend appears where the CMCs are less cytotoxic but have a much better selectivity index in breast cell lines (MCF10A/MCF-7) (Figure 5). The average GI_50_ value of all cell lines excluding MCF10A was plotted against the ctDNA binding affinity; however, no correlation was evident. Cytotoxicity, similarly, showed no correlation with fluorescence or lipophilicity. This echoes our findings with 56MESS type complexes which have excellent ctDNA binding properties, but their binding affinity does not correlate to their cytotoxicity.

The difference in cytotoxicity between the CMCs and non-cyclometallated analogues suggests that these complexes do not have the same mechanism of action, and thus the CMC cannot be used to investigate the mechanism of action of 56MESS type complexes. However, between both the increased selectivity index (indicating that they may be less toxic to non-cancerous cells) and the ability to utilise their inherent fluorescence, these complexes provide an exciting new avenue of research for potential anticancer agents.

## 3. Materials and Methods

### 3.1. Materials and Preparation

Reagents were used as received unless otherwise specified. All solvents used were of analytical grade or higher and purchased from Labserv (Mandaluyong, Philippines), Chem-Supply (Gillman, SA, USA), or Merck Chemicals (Darmstadt, Germany). Potassium tetrachloroplatinate (K_2_PtCl_4_) was purchased from Precious Metals Online. 2-phenylpyridine, benzo(h)quinoline, dibenzo(f,h)quinoline, (1*S*,2*S*)-(+)-1,2-diaminocyclohexane, and (1*R*,2*R*)-(+)-1,2-diaminocyclohexane were purchased from Sigma-Aldrich. Methanol, ethanol, diethyl ether, and methoxy-ethanol were obtained from Honeywell. Deuterated solvents d_6_-dimethylsulphoxide (DMSO-d_6_, 99.9%) and deuterium oxide (D_2_O, 99.9%) were purchased from Cambridge Isotope Laboratories (Tewksbury, MA, USA).

### 3.2. Synthesis

#### 3.2.1. Synthesis of [Pt(C_L_)(Cl)_2_]^−^

Potassium tetrachloroplatinate (242.83 mg:585.00 mmol:1 equiv.) was dissolved in a 3:1 solution of water and methoxy-ethanol. Then, the cyclometallated ligand (C_L_), Benzo(h)quinoline (263.65 mg:1.471 mol:2.5 equiv.) was added to the solution before being heated to 80 °C, at which it was held for 16 h whilst stirring. After 16 h, the insoluble [Pt(bequ)(Cl)_2_]^−^ was synthesised and could be filtered out of the reaction solution and washed with 3 mL of diethyl ether. This synthesis was repeated using dibenzo(f,h)quinoline and 2-phenylpyridine as the C_L_.

#### 3.2.2. Synthesis of [Pt(DACH)(C_L_)]^+^ Complexes

[Pt(C_L_)Cl_2_] (1.1 equiv.) and either (1*S*,2*S*)-(+)-1,2-diaminocyclohexane or (1*R*,2*R*)-(+)-1,2-diaminocyclohexane (1 equiv.) were refluxed for 12 h, resulting in the solution transforming from an opaque pale dark brown to a clear dark orange solution. The volume was then reduced to allow purification via precipitation using a combination of methanol and diethyl ether. At this point, the complex would typically be quite pure (according to HPLC), but if the reaction was left too long or if residue was not properly stirred into the solution during reflux, impurities that could not be removed by precipitation would form. If this occurred, the solution could be further purified using a Vac 20cc (5 g) C_18_ Sep-Pak^©^ column connected to a pump apparatus with a UV detector (Bio-Rad (Hercules, CA, USA), EM-1 Econo™ UV Monitor). The column was activated with methanol (20 mL) and then flushed with water (~40 mL) until the UV absorbance equilibrated. The purified solution was then reduced under vacuum and freeze-dried (>95% purity achieved for all complexes) (Table 1).

### 3.3. Cytotoxicity Methodology

Cytotoxicity assay studies were performed at Calvary Mater Newcastle Hospital, Waratah, NSW, Australia. In vitro studies were performed according to described methods [37]. Complexes were prepared in DMSO as stock treatment (30 mM) solutions and stored at −20 °C. All cell lines were cultured in a humidified atmosphere with 5% CO_2_ at 37 °C and maintained in Dulbecco’s modified eagle’s medium (DMEM; Trace Biosciences, Melbourne, Australia) supplemented with 10% foetal bovine serum, sodium bicarbonate (10 mM), penicillin (100 IU mL^−1^), streptomycin (100 μg mL^−1^), and *L*-glutamine (4 mM). The non-cancer MCF10A cell line was cultured in DMEM.F12 (1:1) cell culture media (Composition in SI). Cytotoxicity was determined by plating cells in duplicate in 100 μL medium at a density of 2500–4000 cells per well in 96-well plates. After 24 h, when cells were in logarithmic growth, media (100 μL) with or without the test agent were added to each well (day 0). After 72 h of exposure, growth inhibitory effects were evaluated by MTT (3-[4,5-dimethylthiazol-2-yl]-2,5-diphenyltetrazolium bromide) assay, and absorbance was read at 540 nm. An eight-point dose–response curve was produced, from which the drug concentration at which cell growth is inhibited by 50% (GI_50_) was calculated. These calculations were based on the difference between the optical density values on day 0 and those at the end of drug exposure.

### 3.4. Biophysical Characterisation

NMR spectral data were obtained using a 400 MHz Bruker Avance spectrometer at 298 K, using 10 mm samples prepared in D_2_O. ^1^H NMR spectra were obtained using a spectral width of 8250 Hz and 65,536 data points, while ^195^Pt NMR spectra were acquired using a spectral width of 85,470 Hz and 674 data points. ^1^H-^195^Pt HMQC spectra were recorded using a spectral width of 214,436 Hz and 256 data points for the ^195^Pt nucleus (F1 dimension) and a spectral width of 4808 Hz with 2048 data points for the ^1^H nucleus (F2 dimension).

UV spectra were recorded on a Cary 3500 UV–Vis spectrophotometer at room temperature in the 200–400 nm range, using a 10 mm quartz cell. All samples were automatically corrected for solvent baseline. The titration of a stock solution into a known volume of solvent in triplicate allowed the calculation of the extinction coefficient. Additionally, the spectrum of each CMC at 30 µM in in a 40 mM K_2_HPO_4_/KH_2_PO, 10 mM KCl buffer at pH 7.0 was initially recorded before the titration of a 6.5 µM stock solution of ctDNA. Upon the addition of each aliquot of DNA, the solution was gently mixed and allowed to stand for 2 min before spectra were obtained to allow time for the CMC to interact with the DNA. Experiments were performed in triplicate for each CMC.

HPLC were collected on Agilent Technologies 1260 Infinity machine equipped with a Phenomenex Onyx™ Monolithic C18 reverse phase column (100 × 4.6 mm, 130 Å). The mobile phase comprised 0.06% TFA in water (solvent A) and 0.06% TFA in ACN:H_2_O (90:10, solvent B). Complexes were dissolved in water and flowed at a 0–100 gradient over 15 min, with an additional 15 min flush between each sample.

Electrospray ionisation mass spectroscopy (ESI-MS) experiments were performed using a Waters TQ-MS triple quadrupole mass spectrometer in the positive mode. Sample solutions were made up to 0.5 mM in H_2_O and flowed at 0.1 mL/min. The desolvation temperature of 300 °C and desolvation flow rate (nitrogen) of 500 L/hr remained consistent, whilst the cone voltage and capillary voltage were varied for each sample to adjust for fragmentation. Spectra were collected over varied *m/z* ranges depending on the target mass.

Luminescence quantum yields were obtained using the comparison method, where [Ru(bpy)_3_]Cl_2_ was the standard chosen due to its similar excitation and emission maxima [38]. Dilute solutions were prepared and titrated into water and the UV spectra were recorded, ensuring that they absorbed at no more than 0.1 abs at 310 nm after 5 titres. The solution was then titrated again into water, and the fluorescence emission, when excited at 310 nm, was recorded. This was repeated three times per sample, and the results were then graphed to discover the final QY value.

Fluorescent intercalator displacement (FID) assays were obtained using the same instrument and by using 10 mm quartz cells. Stock solutions CMCs 1–3 (30 µM) were titrated into a solution of 75 µL ethidium bromide, 150 µM ctDNA in a 40 mM K_2_HPO_4_/KH_2_PO, and 10 mM KCl buffer at pH 7.0. The fluorescence was measured from 550 to 750 nm excited at 480 nm and inverting 4 times and waiting 3 min between the addition of ctDNA and scan time to allow adequate time for the CMCs to interact with the DNA.

The direct fluorescent measurements of DNA binding constants were also obtained with the same fluorimeter using 10 mm path length quartz cells. A 0.065 M stock solution of ctDNA was titrated into a 10 µM solution of CMCs 1–3 in a 40 mM K_2_HPO_4_/KH_2_PO, 10 mM KCl buffer at pH 7.0. The fluorescence was measured from 550 to 750 nm excited at 320 nm inverting 4 times and waiting 3 min between the addition of ctDNA and scan time to allow adequate time for the CMCs to interact with the DNA.

Lipophilicity was calculated using a RP-HPLC method to determine log Kw, whereby a stock solution was injected at different isocratic ratios ranging from 70 to 90% solvent B (organic) at a flow rate of 1 mL min^−1^. These experiments were undertaken on an Agilent Technologies 1260 Infinity machine equipped with a Phenomenex Onyx™ Monolithic C18 reverse phase column (100 × 4.6 mm, 130 Å). The mobile phase comprised 0.06% TFA in water (solvent A) and 0.06% TFA in ACN:H_2_O (90:10, solvent B). The dead zero volume time was determined using potassium iodide as an external dead volume marker, further details are provided in the Supplementary Materials.

CD spectra were obtained using a Jasco-810 spectropolarimeter at room temperature. The instrument was left to equilibrate for 30 min prior to use. Spectra were obtained in a 10 mm quartz cell and were measured from 400 to 200 nm with a data pitch of 1 nm, bandwidth of 1 nm, and response time of 1 s. For each spectrum, 40 accumulations were collected, and a water baseline was subtracted. Additional CD experiments were undertaken where 1 and 2 equivalents of ctDNA were added to a cuvette containing one equivalent of CMC that was baseline corrected to avoid interference from the chiral centres of the complexes.

## 4. Conclusions

To summarise, six novel resolved chiral cyclometallated complexes (CMCs) were successfully synthesised, and preliminary testing was undertaken to discover if these complexes could be utilised as anticancer agents. The design of the CMCs was inspired by a group of complexes previously synthesised by our group, which have excellent cytotoxicity but could be more selective towards cancer cells. The CMCs have a similar cytotoxicity to cisplatin, and a significantly higher selectivity index than their non-cyclometallated analogues. Three of the six CMCs had a selectivity index over two in breast cell lines (MCF10A/MCF-7), indicating that they may be more selective towards cancer cells, and hence reducing toxicity to healthy cells. It was hypothesised that the complexes would bind to DNA and that this could be what leads to their cytotoxicity. DNA binding affinity was investigated using fluorescent intercalator displacement studies as well as using the CMCs inherent fluorescence to monitor DNA binding directly. Both methods found that all CMCs had good DNA binding properties based on Ka, and the results suggest that this direct measurement may be more accurate than the more commonly used FID experiment. These complexes are promising as potential anticancer agents, and our future work will focus on the location of cellular accumulation and the digestion of the CMC to shed light on their mechanism of action.

## 5. Patents

The complexes described in this manuscript are included in Western Sydney University 2022 Australia, “Platinum (IV) complexes” 197461PRV and its accompanying international PCT application no. PCT/AU2023/050027 completed in 2023.

## Figures and Tables

**Table 1:** Summary of synthetic yield, ESI-MS, lipophilicity, and extinction coefficients of complexes **1**–**3**.

Complex	Yield (%)	ESI-MS (*m*/*z*)	Lipophilicity × 10^−2^	UV/*λ*_max_ (nm) (ε/mol^−1^·dm^3.^cm^−1^) × 10^2^
		[M]^+^ Calc. (Found)		
**1a**	78.9	463.15 (463.1557)	1.41 ± 0.0049	241 (213 ± 1.07), 287 (75 ± 0.42)
**2a**	78.32	487.15 (487.1436)	2.06 ± 0.097	241 (129 ± 1.71), 272 (173 ± 0.80)
**3a**	79.36	537.16 (538.1541)	0.77 ± 0.032	256 (216 ± 0.69), 235 (27 ± 0.74)
**1b**	74.23	463.15 (463.1463)	1.52 ± 0.063	241 (358 ± 1.11), 287 (128 ± 0.36)
**2b**	77.95	487.15 (487.1436)	3.97 ± 0.053	241 (173 ± 0.71), 272 (53 ± 0.21)
**3b**	73.26	537.16 (538.1475)	0.4 ± 0.029	256 (303 ± 1.93), 235 (155 ± 1.41)

**Table 2:** Fluorescent properties of complexes **1**–**3**.

Complex	Ex_max_	Em_max_	Φ_F_
**1a**	287.96	486.00	0.160
**2a**	262.03	483.03	0.218
**3a**	262.03	361.07	0.176
**1b**	338.00	484.84	0.103
**2b**	378.00	524.02	0.187
**3b**	300.00	361.07	0.315

**Table 3:** Summary of synthetic yield, ESI-MS, lipophilicity, and extinction coefficients of complexes **1**–**3** (the best results highlighted in the table).

Complex	Point of Experimental Stoichiometry of Binding	ΔFsat	Ka × 10^4^	n	Kf	Kq	KSV
**1a**	2.84	83.82	2.75	0.778	4.35	0.329	1.988
**1b**	2.65	86.38	1.58	0.757	4.23	0.328	1.957
**2a**	2.52	89.29	1.58	0.592	3.25	0.241	1.339
**2b**	2.47	89.01	1.59	0.668	3.78	0.271	1.662
**3a**	2.38	91.83	1.07	0.579	3.18	0.237	1.342
**3b**	2.62	82.38	3.98	0.757	4.22	0.297	1.727

**Table 4:** Binding affinity of CMCs to DNA according to inherent fluorescence method (the best results highlighted in the table).

	Ka × 10^4^	R^2^
**1a**	2.20	0.997
**1b**	1.46	0.994
**2a**	1.11	0.997
**2b**	1.39	0.995
**3a**	1.11	0.998
**3b**	3.68	0.953

**Table 5:** GI_50_ data for complexes **b** compared to cisplatin.

	GI_50_ Concentration (µM) That Inhibits Cell Growth by 50%							
	1a	1b	2a	2b	3a	3b	Cisplatin	56MESS
HT29 Colon	13 ± 1.0	41 ± 2.7	16 ± 1.3	30 ± 2.3	16 ± 0.00	16 ± 0.3	11.3 ± 1.9	nd
U87 Glioblastoma	5.3 ± 0.3	23 ± 2.2	8.6 ± 1.1	4.3 ± 0.3	16 ± 0.6	4.1 ± 0.3	3.8 ± 1.1	0.076 ± 0.014
MCF-7 Breast	8.4 ± 2.0	13 ± 1.7	5.4 ± 1.0	7.7 ± 2.0	15 ± 0.5	4.2 ± 0.3	6.5 ± 0.8	0.050 ± 0.020
A2780 Ovarian	12 ± 0.7	31 ± 2.9	18 ± 2.3	5.0 ± 0.3	12 ± 0.9	4.5 ± 0.3	1 ± 0.1	0.030 ± 0.004
H460 Lung	5.8 ± 0.4	25 ± 2.9	11 ± 1.5	8.1 ± 1.0	16 ± 0.9	8.2 ± 2.9	0.9 ± 0.2	0.037 ± 0.009
A431 Skin	4.2 ± 0.5	29 ± 2.6	12 ± 2.4	3.4 ± 0.3	14 ± 0.6	3.9 ± 0.4	2.4 ± 0.3	0.051 ± 0.021
Du145 Prostate	9 ± 2.0	41 ± 0.9	26 ± 2.0	20 ± 2.7	23 ± 2.1	16 ± 1.5	1.2 ± 0.1	0.007 ± 0.002
BE2-C Neuroblastoma	34 ± 7.5	38 ± 4.6	23 ± 3.5	48 ± 1.7	9 ± 1.3	33 ± 1.2	1.9 ± 0.2	0.10 ± 0.016
SJ-G2 Glioblastoma	11 ± 0.7	20 ± 4.8	13 ± 2.2	10 ± 2.2	17 ± 0.6	10.1 ± 2.6	0.4 ± 0.1	0.074 ± 0.018
MIA Pancreas	8 ± 1.6	41 ± 2.0	20 ± 3.0	8.1 ± 1.6	15 ± 0.7	12.5 ± 2.0	7.5 ± 1.3	0.015 ± 0.002
MCF10A Breast (normal)	6.4 ± 0.5	32 ± 2.7	20 ± 1.0	10.3 ± 1.9	16 ± 0.6	8.5 ± 1.4	nd	0.032 ± 0.007
ADDP Cis Res Ovarian	5.1 ± 0.3	26 ± 0.6	14 ± 0.3	19 ± 3.0	15 ± 0.00	6.9 ± 0.4	nd	nd
SI	0.76	2.46	3.70	1.33	1.07	2.02	nd	0.64

nd = no data available. SI = GI_50_ for normal cells/GI_50_ for cancer cells (MCF10A/MCF-7). SI value indicates selectivity towards cancer cells > SI ≥ selectivity. The key findings are highlights in the table.

## Data Availability

The data presented in this study are available within the article and Supplementary Materials (Table S1 and Figures S1–S59).

## Supplementary Materials

The following supporting information can be downloaded at https://www.mdpi.com/article/10.3390/ijms24098049/s1. NMR spectra, HPLCs, CD, Lipophilicity results and extinction coefficients. References [39,40,41] are cited in the supplementary materials.
