# Supplementary material for: Synthesis and Characterisation of Fluorescent Novel Pt(II) Cyclometallated Complexes with Anticancer Activity
Source: Int J Mol Sci. 2023 Apr 28;24(9):8049. doi: 10.3390/ijms24098049 (PMC10178562; doi:10.3390/ijms24098049)
Supplement: Supplementary file 1 [file ijms-24-08049-s001.zip › ijms-2344389-supplementary.pdf]

Supplementary information

Contents

Supplementary information 1
NMR 3
[Pt(Pepy)(SSDACH)]$^+$ 4
[Pt(Pepy)(RRDACH)]$^+$ 5
[Pt(Bequ)(SSDACH)]$^+$ 6
[Pt(Bequ)(RRDACH)]$^+$ 7
[Pt(DiBequ)(SSDACH)$^{]+}$ 8
[Pt(DiBequ)(RRDACH)]$^+$ 9
Extinction coefficients 10
[Pt(Pepy)(SSDACH)]$^+$ 10
[Pt(Pepy)(RRDACH)]$^+$ 11
[Pt(Bequ)(SSDACH)]$^+$ 11
[Pt(Bequ)(RRDACH)]$^+$ 12
[Pt(DiBequ)(SSDACH)$^{]+}$ 12
[Pt(DiBequ)(RRDACH)]$^+$ 13
UV Spectra 13
Ligands 13
Complexes 14
Lipophilicity 15
CD 17
[Pt(Pepy)(DACH)]$^+$ 17
[Pt(Bequ)(DACH)]$^+$ 17
[Pt(DiBequ)(DACH)$^{]+}$ 18
HPLC 19
[Pt(Pepy)(SSDACH)]$^+$ 19
[Pt(Pepy)(RRDACH)]$^+$ 19
[Pt(Bequ)(SSDACH)]$^+$ 20
[Pt(Bequ)(RRDACH)]$^+$ 20
[Pt(DiBequ)(SSDACH)$^{]+}$ 21
[Pt(DiBequ)(RRDACH)]$^+$ 21
ESI-MS 22
[Pt(Pepy)(SSDACH)]$^+$ 22

[Pt(Pepy)(RRDACH)]$^+$ ... 22
[Pt(Bequ)(SSDACH)]$^+$ ... 23
[Pt(Bequ)(RRDACH)]$^+$ ... 24
[Pt(DiBequ)(SSDACH)$^{]+}$ ... 24
[Pt(DiBequ)(RRDACH)]$^+$ ... 25
Fluorescence ... 27
QY ... 27
DNA binding ... 27
UV ... 27
Inherent Fluorescence ... 30
Fluorescent Intercalation Displacement Assay ... 33
FID vs Inherent fluorescence ... 36
Cytotoxicity ... 37
References ... 38

NMR

NMR Spectral data were obtained using a 400 MHz Bruker Avance spectrometer at 298 K, using samples prepared in D_2O. ^1H NMR spectra were obtained using a spectral width of 8250 Hz and 65536 data points, while ^{195}Pt NMR spectra were acquired using a spectral width of 85470 Hz and 674 data points. Chemical shifts are reported in parts per million (ppm) with J coupling reported in Hz.

Firstly, the peaks were integrated, as we would expect the peaks in aromatic region had an integration of 2 and the protons in the aliphatic region had an integration of 2 the have a value of two as the ligand is asymmetric so none of the peaks are perfectly merged. In some cased the peaks overlapped in which case they were labelled by the process of elimination based on the other peaks. Next, we looked at the J coupling, the duplex peak assigned to P2/9 had a larger j coupling than duplex the peak assigned to the P4/7 confirming our assignment (for 3a/b these were 2/11 and 4/9). We see that these peaks can be found further upstream or downstream to each other depending on the concentration of the sample which we believe is due to π- π stacking. Next, we identified the 5/6 (6/7 for 3a/b) peaks these could easily be identified in the COSY as only coupling to each other. Next the 3/8 protons were assigned, we did this again using the cosy, they were identified as the protons who were coupling with two other peaks, (which we can thus identify as the 9/2 and 4/7 peaks). This left us to identify the remaining peaks bases on the spitting pattern, 3/8 and 4/7 having triplets rather than doublets like the other peaks. Where the assignment was less clear we incorporated the knowledge gained from the HMQC which tells us which protons are closely coupled to the Pt centre. For the assignment of the protons in the aliphatic region we used the same methodology as in our previously published papers however in this case we see each peak is duplicated due to the asymmetry of the cyclometallated ligand. All proton assignments are summarised in table 1 bellow.

Table S1: NMR assignments (ppm) for complexes 1-3 including J coupling (Hz) and integration(H).

Proton	Complex					
	1a	1b	2a	2b	3a	3b
2	δ ~7.19(merged 2,3 and 7, H3)	δ ~7.16(merged 2,3 and 7, H3)	δ 7.71 (d, J= 8.09 Hz, H1)	δ 7.69 (d, J=7.96 Hz, H1)	δ 7.69(merged 2 and 7, H2)	δ 7.79 (merged 2 and 10 H2)
3			δ 7.62(t, J= 8.66 Hz, H1)	δ 7.56(t, J= 8.38 Hz, H1)	δ 7.56 (m, H1)	δ 8.40 (t, J= 4.22 Hz, H1)
4	δ 7.95 (t, J= 8.05 Hz, H1)	δ 7.92 (t, J= 7.99 Hz, H1)	δ 7.39 (merged 4 and 6, H2)	δ 7.36 (d, J=7.54 Hz, H1)	δ 7.82 (d, J=8.37 Hz, H1)	δ 9.26 (d, J=7.90 Hz, H1)
5	δ 7.83 (d, J= 7.98 Hz, H1)	δ 7.78 (d, J= 8.26 Hz, H1)	δ 7.79 (d, J= 8.26 Hz, H1)	δ 7.76(d, J= 8.80 Hz, H1)	δ 8.68 (d, J= 7.95 Hz, H1)	δ 9.44 (d, J= 8.43 Hz, H1)
6	δ 7.62 (d, J= 7.26 Hz, H1)	δ 7.36 (d, J= 8.26 Hz, H1)	δ 7.39 (merged 4 and 6, H2)	δ 7.31 (m, H2)	δ 7.69(merged 2 and 7, H2)	δ 8.85 (merged 6 and 8 H2)
7	δ ~7.19 (merged 2,3 and 7, H3)	δ ~7.16(merged 2,3 and 7, H3)	δ 8.38 (merged 7 and 9, H2)	δ 8.28 (merged 7 and 9, H2)	δ 7.69(merged 2 and 7, H2)	δ 7.58 (d, J= 4.74 Hz, H1)
8	δ 7.51 (t, J= 7.71 Hz, H1)	δ 7.49 (m, H1)	δ 7.52 (t, J= 7.80 Hz, H1)	δ 7.51 (t, J= 7.54 Hz, H1)	δ 7.89 (merged 8 and 11 H2)	δ 8.85 (merged 6 and 8 H2)
9	δ 8.30 (d, J= 5.78 Hz, H1)	δ 8.24 (d, J= 6.00 Hz, H1)	δ 8.38 (merged 7 and 9, H2)	δ 8.28 (merged 7 and 9, H2)	δ 7.69(merged 2and 7, H2)	δ 9.02 (d, J= 4.22 Hz, H1)
10					δ 7.60 (t, J= 7.12 Hz, H1)	δ 7.79 (merged 2 and 10 H2)
11					δ 7.89 (merged 8 and 11 H2)	δ 9.22 (d, J= 7.38 Hz, H1)
1′/2′	δ 2.41(m, H2)	δ 2.30(m, H2)	δ 2.73(m, H2)	δ 2.73(m, H2)	δ 2.45(m, H2)	δ 2.33 (m, H2)
3′/6′	δ 2.07(d, J= 13.16 Hz, H2)	δ 2.03(dd, J= 13.64 Hz, H2)	δ 2.34(m, H2)	δ 2.31(d, J= 8.67 H2)	δ 2.23(d, J= 9.07 H2)	δ 2.09 (m, H2)
3′/6′	δ 1.60 (d, J= 7.52 Hz, H2)	δ 1.56 (d, J= 7.71 Hz, H2)	δ 2.08 (d, J= 12.23 Hz, H2)	δ 1.99(m, H2)	δ 1.61(d, J=7.32, H2)	δ 1.64 (m, H2)
4′/6′	δ 1.23(m, H2)	δ 1.23(m, H2)	δ 1.23(m, H2)	δ 1.61(m, H2)	δ 1.42(m, H2)	δ 1.43 (m, H2)
4′/6′		δ 1.11(m, H2)	δ 1.25(m, H2)	δ 1.20(m, H2)	δ 1.34(m, H2)	δ 1.21 (m, H2)
^{195}Pt	-3524.3	-3506.6	-3502.2	-3504.1	-3503.0	-3503.0

$[Pt(Pepy)(SSDACH)]^+$

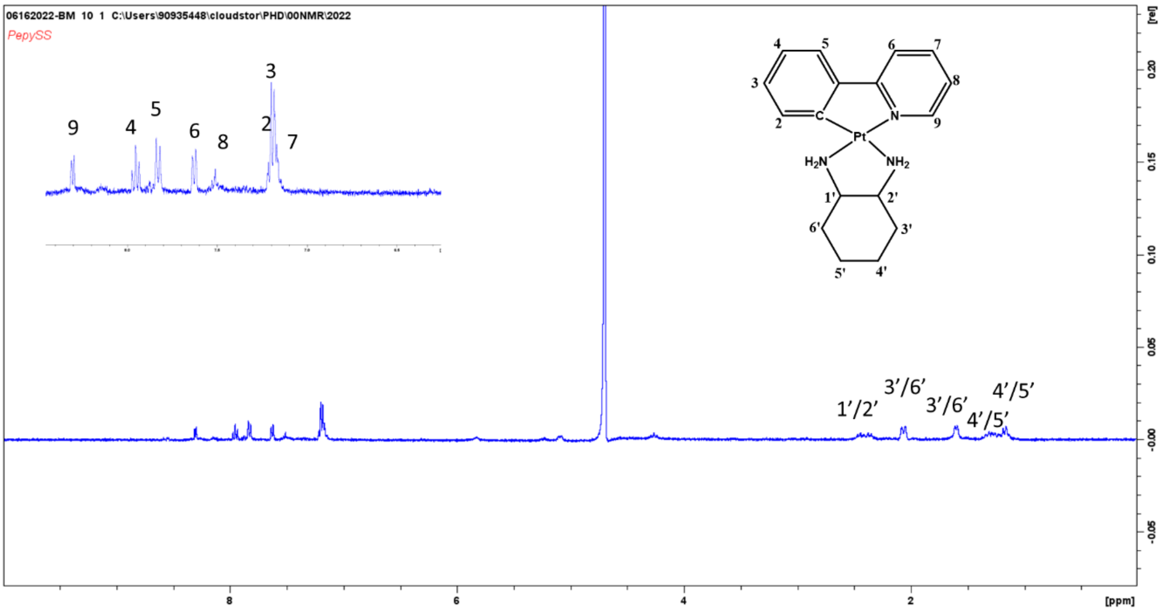

Figure S1: ^{1}H NMR spectra of Pt(II) **1a** in D_2O annotated according to numbered structure top right.

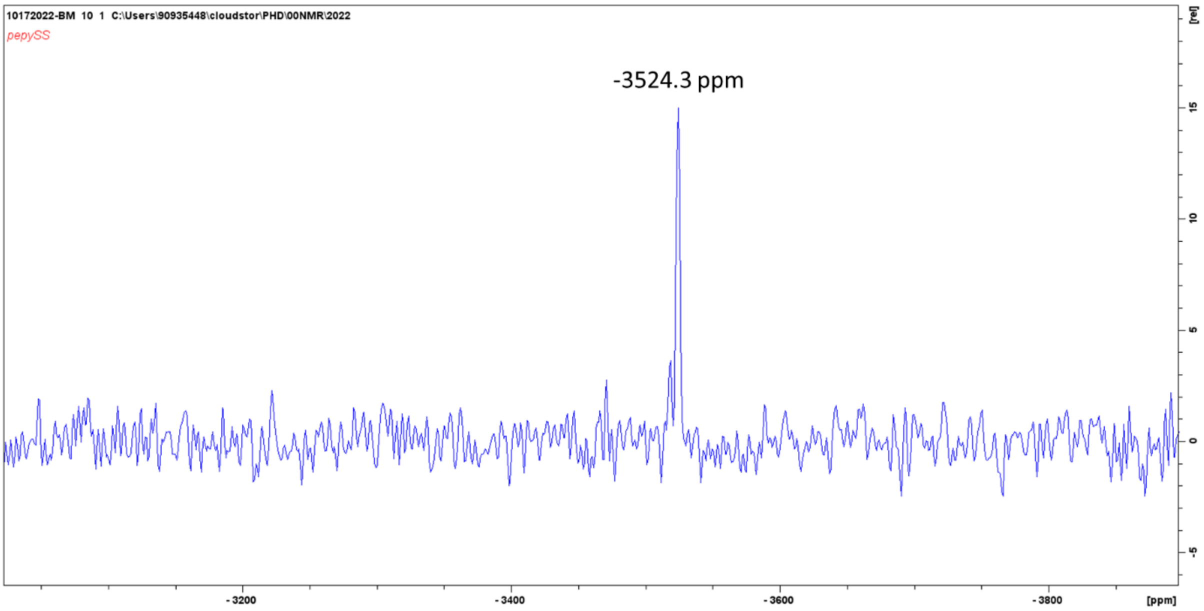

Figure S2: ^{195}Pt NMR spectra of Pt(II) **1a** in D_2O.

[Pt(Pepy)(RRDACH)]$^+$

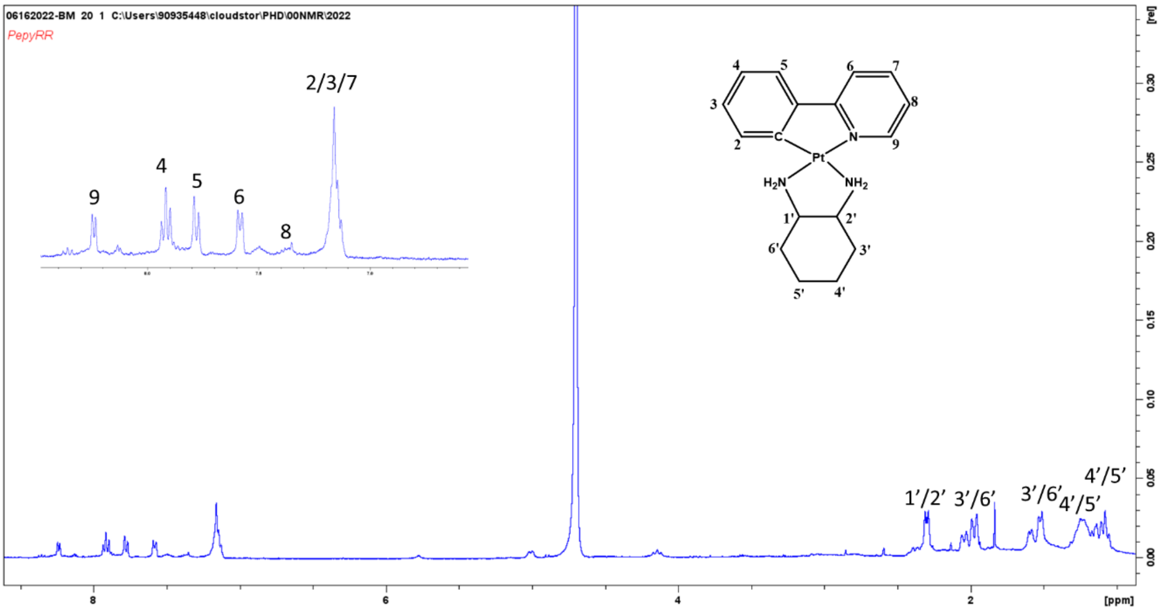

Figure S3: ^{1}H NMR spectra of Pt(II) **1b** in D_2O annotated according to numbered structure top right.

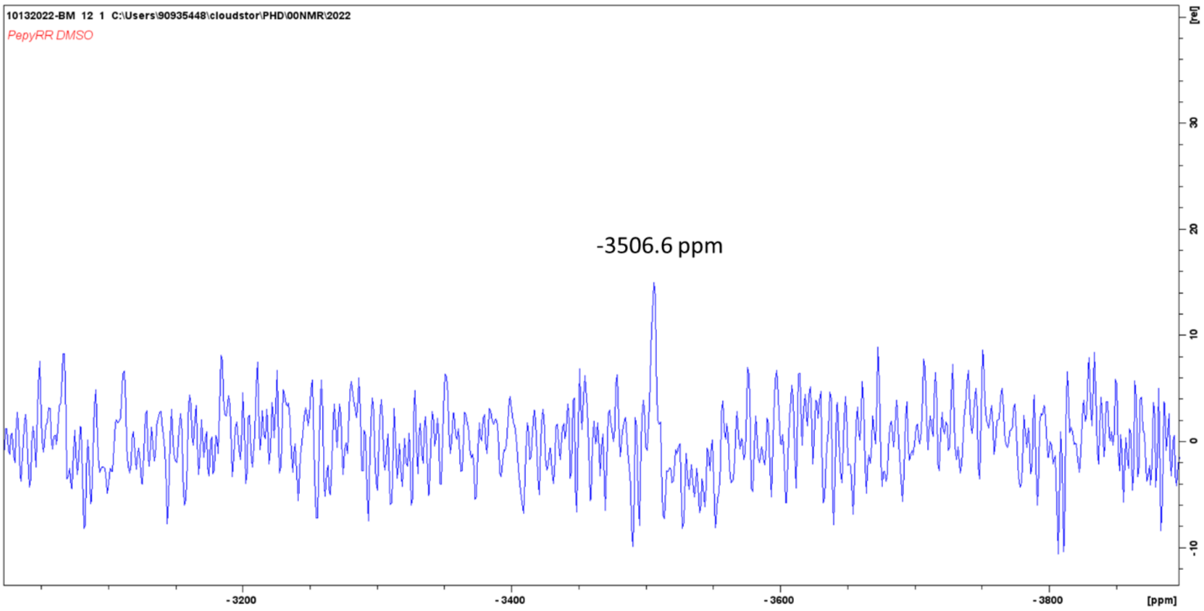

Figure S4: ^{195}Pt NMR spectra of Pt(II) **1b** in D_2O.

[Pt(Bequ)(SSDACH)]$^{+}$

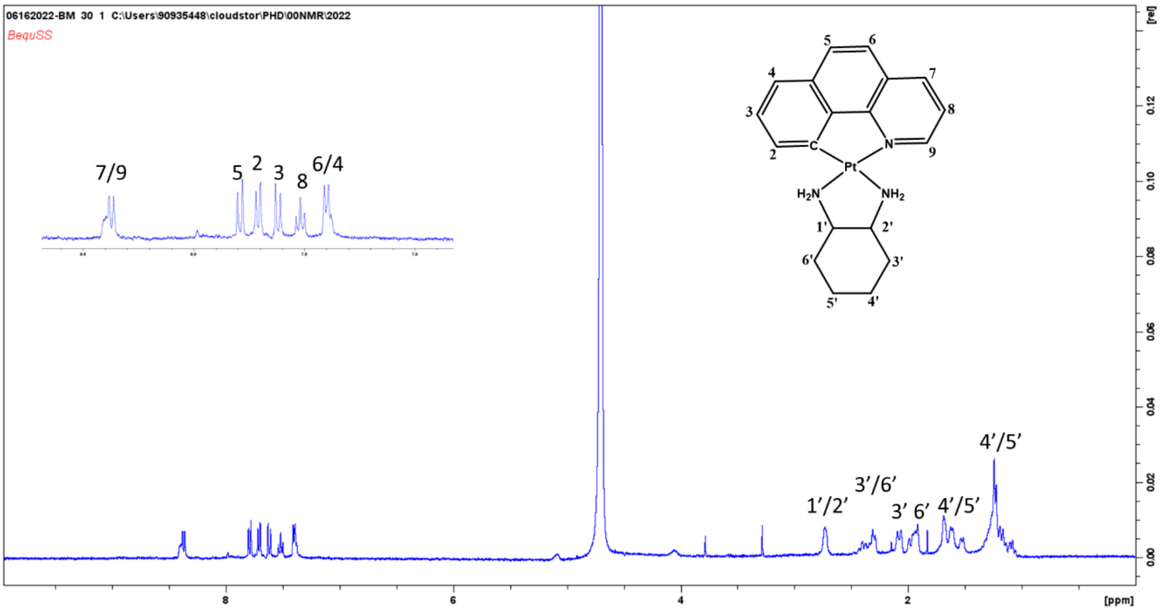

Figure S5: ^{1}H NMR spectra of Pt(II) **2a** in D_2O annotated according to numbered structure top right.

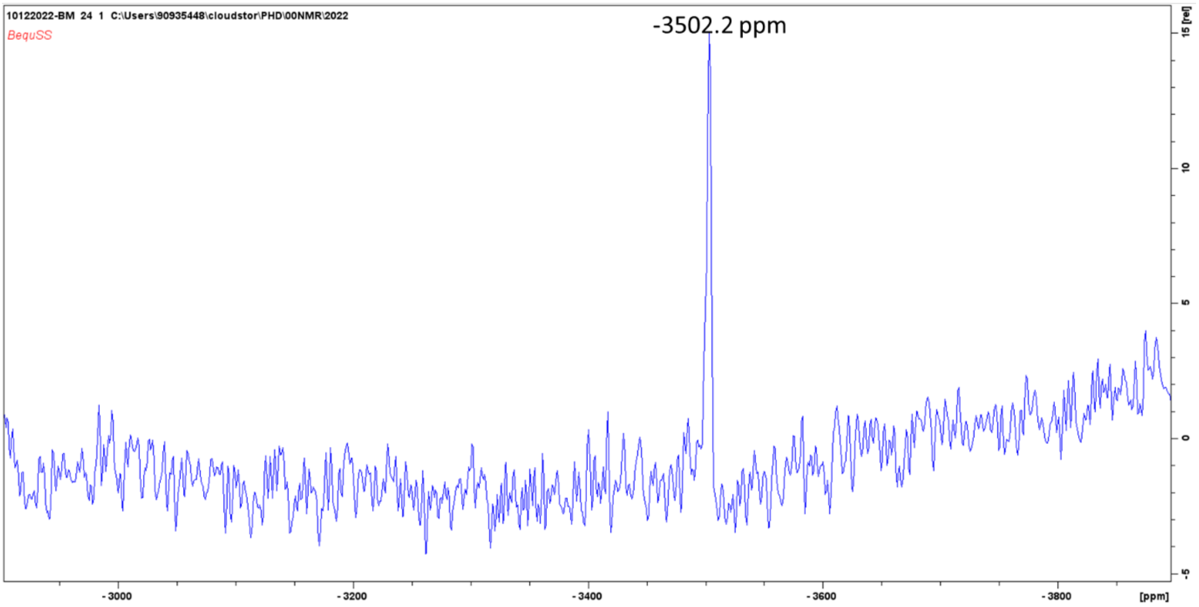

Figure S6: ^{195}Pt NMR spectra of Pt(II) **2a** in D_2O.

[Pt(Bequ)(RRDACH)]$^+$

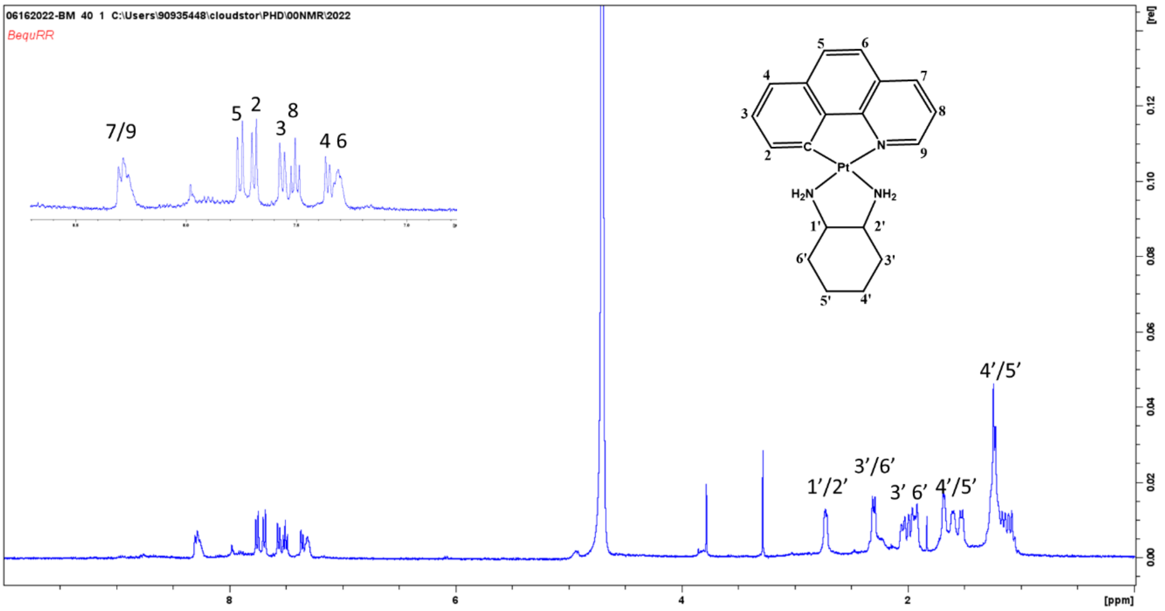

Figure S7: ^{1}H NMR spectra of Pt(II) **2b** in D_2O annotated according to numbered structure top right.

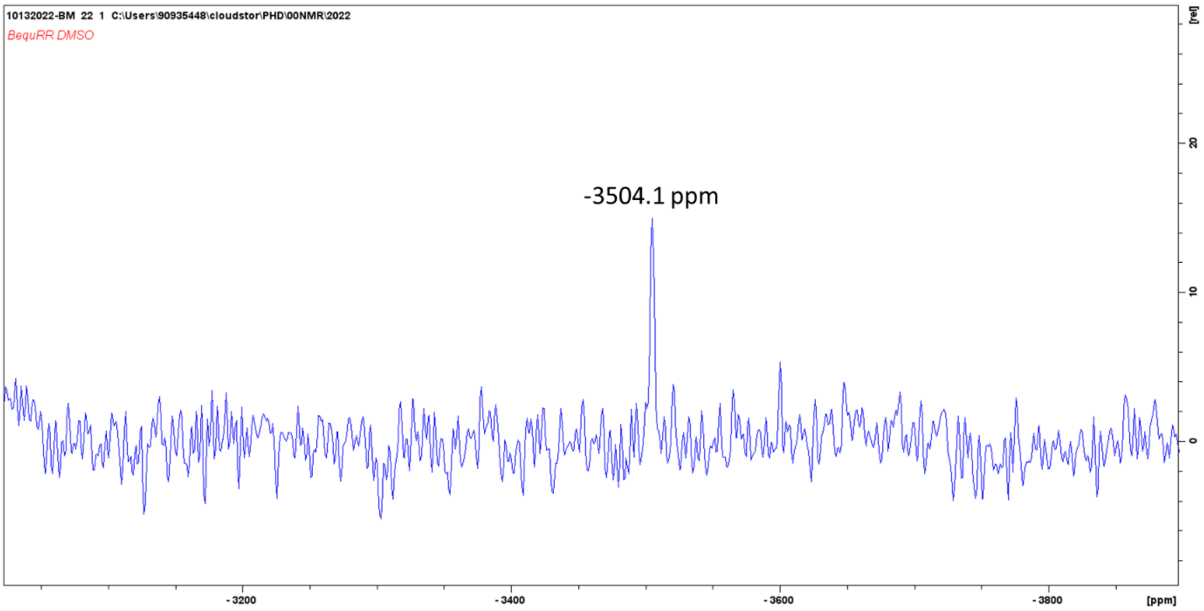

Figure S8: ^{195}Pt NMR spectra of Pt(II) **2b** in DMSO.

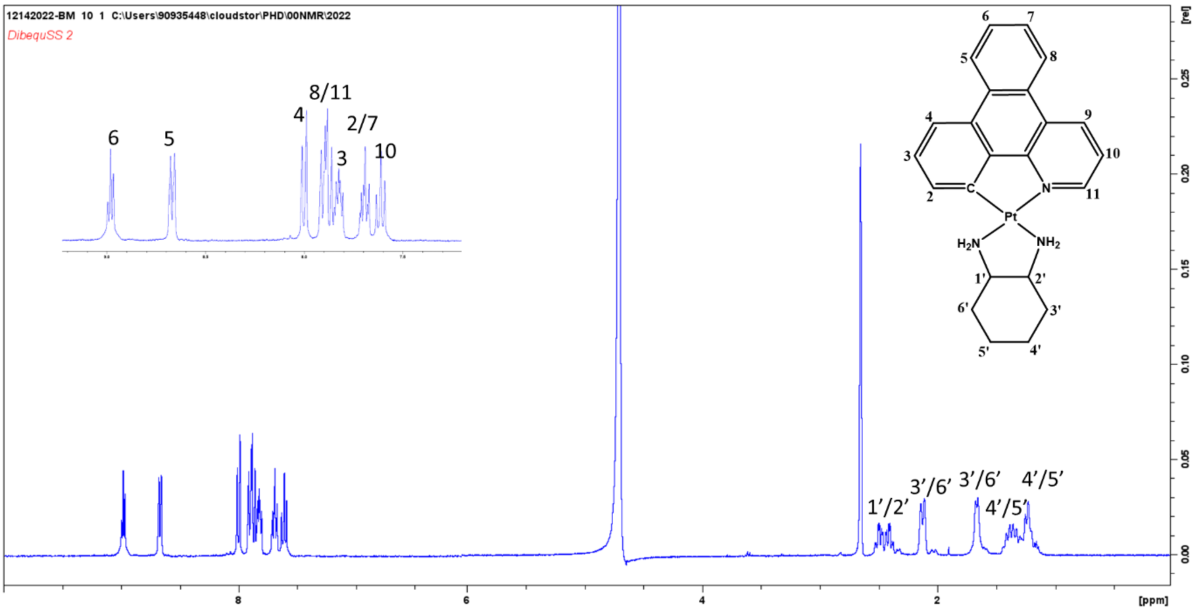

[Pt(DiBequ)(SSDACH)]+

Figure S9: ^{1}H NMR spectra of Pt(II) **3a** in D_2O annotated according to numbered structure top right.

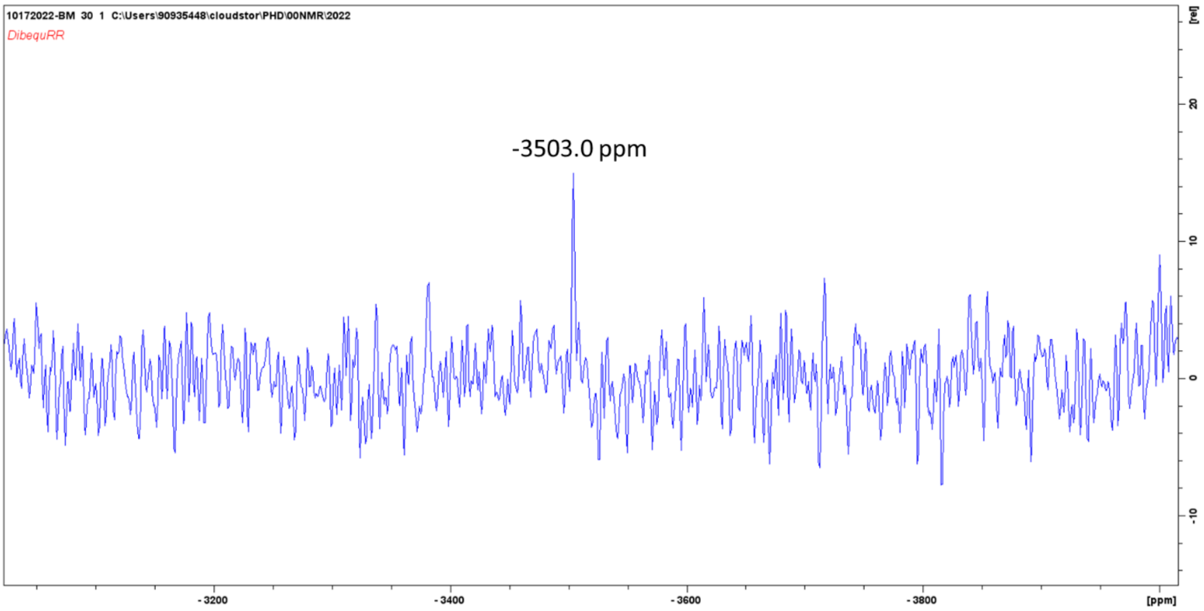

Figure S10: ^{195}Pt NMR spectra of Pt(II) **3a** in D_2O.

[Pt(DiBequ)(RRDACH)]$^+$

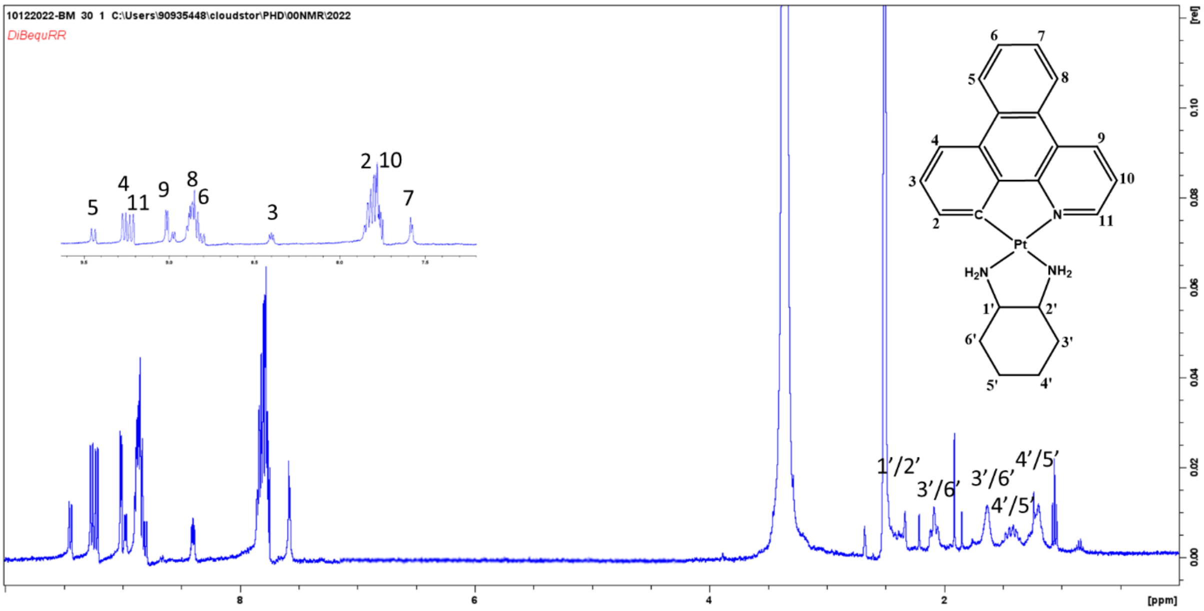

Figure S11: ^{1}H NMR spectra of Pt(II) **3b** in D_2O annotated according to numbered structure top right.

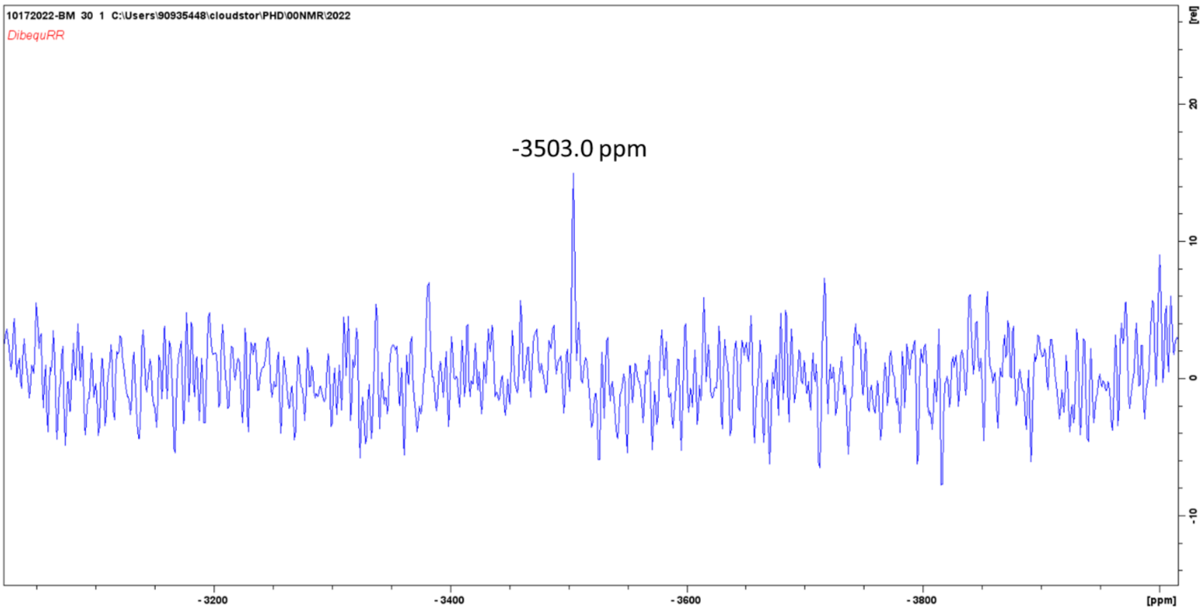

Figure S12: ^{195}Pt NMR spectra of Pt(II) **3b** in D_2O.

Extinction coefficients

[Pt(Pepy)(SSDACH)]$^+$

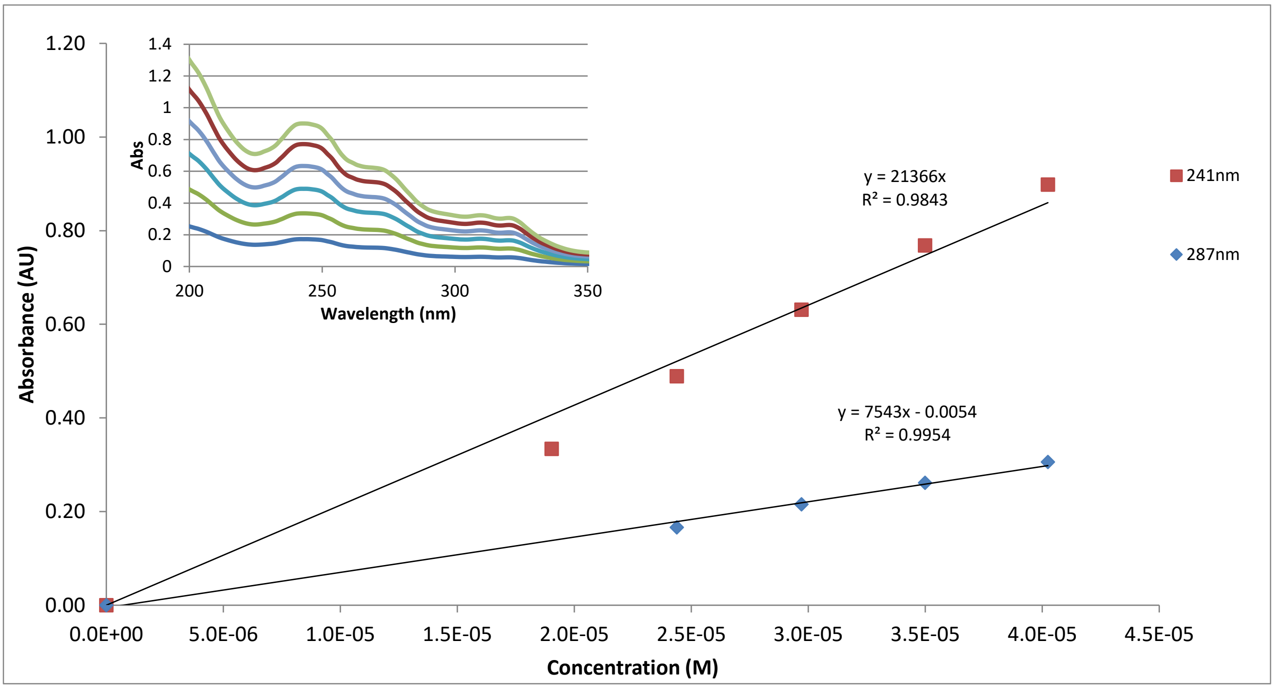

Figure S13: Titration of a stock solution of [Pt(Pepy)(SSDACH)]$^+$ into a known concentration in H_2O and the resulting extinction coefficient calculated based on the two main peaks at 241 nm (red) and 287 nm (blue).

$[Pt(Pepy)(RRDACH)]^+$

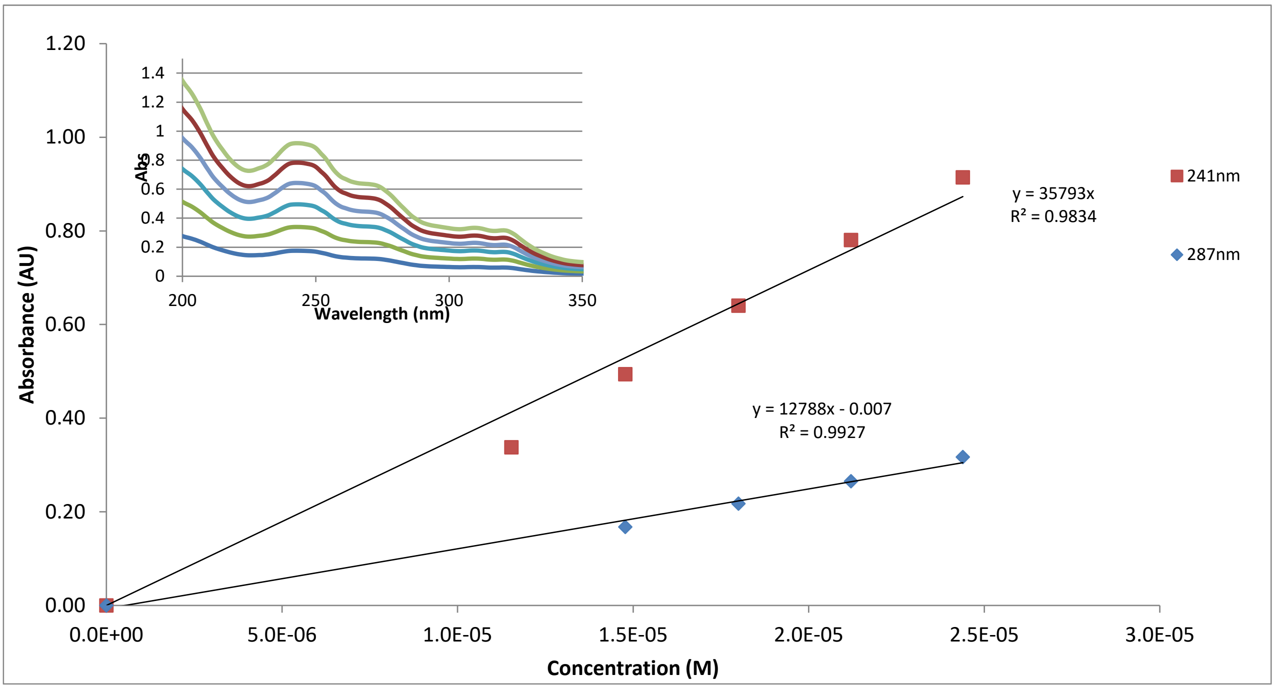

Figure S14: Titration of a stock solution of $[Pt(Pepy)(RRDACH)]^+$ into a known concentration in H_2O and the resulting extinction coefficient calculated based on the two main peaks at 241 nm (red) and 287 nm (blue).

$[Pt(Bequ)(SSDACH)]^+$

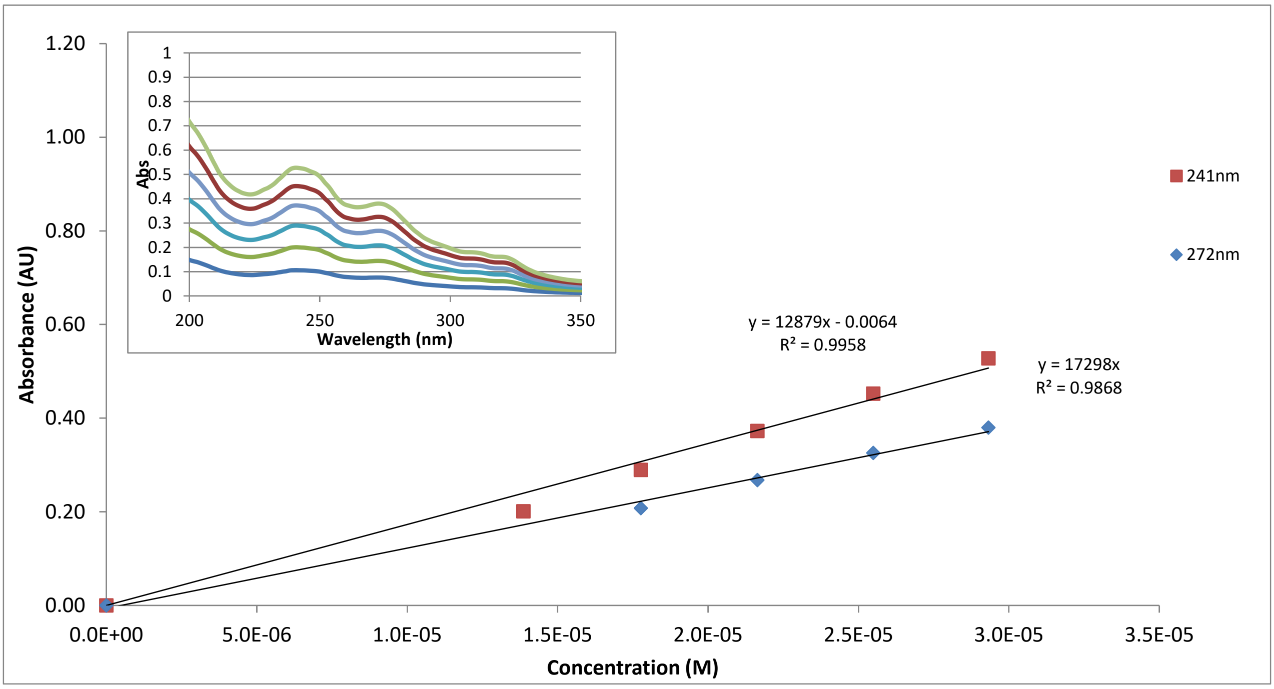

Figure S15: Titration of a stock solution of $[Pt(Bequ)(SSDACH)]^+$ into a known concentration in H_2O and the resulting extinction coefficient calculated based on the two main peaks at 241 nm (red) and 272 nm (blue).

[Pt(Bequ)(RRDACH)]$^+$

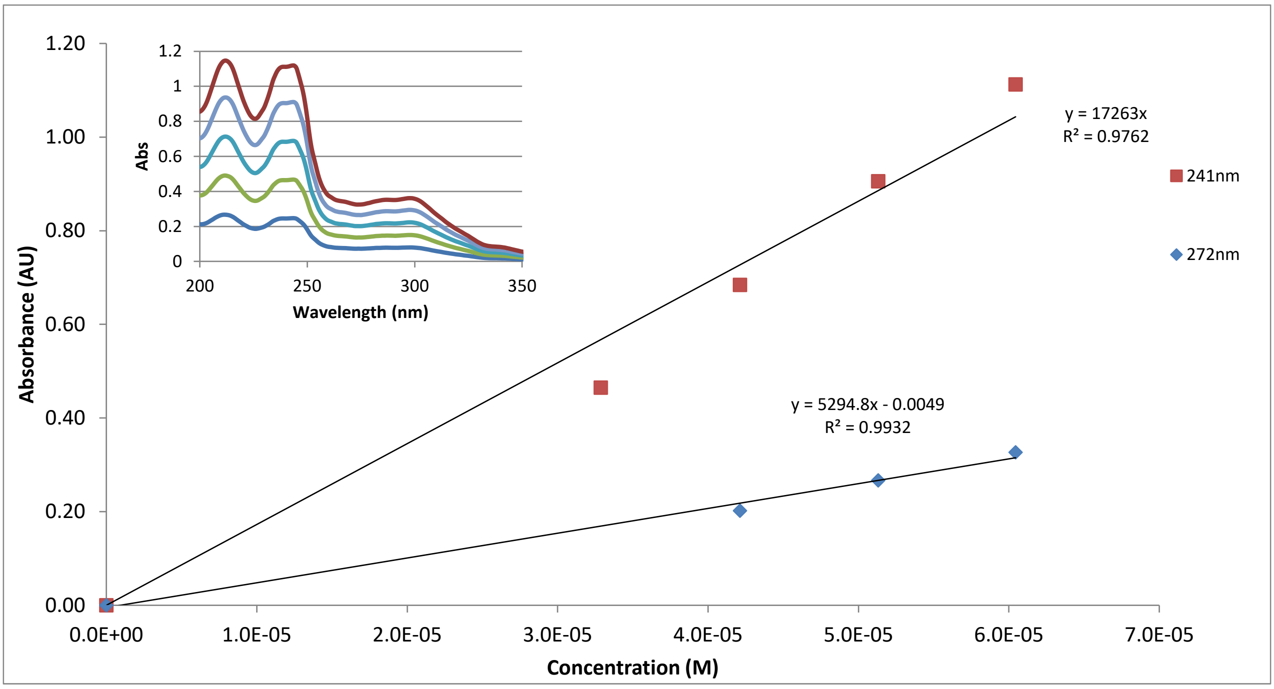

Figure S16: Titration of a stock solution of [Pt(Beqy)(RRDACH)]$^+$ into a known concentration in H_2O and the resulting extinction coefficient calculated based on the two main peaks at 241 nm (red) and 272 nm (blue).

[Pt(DiBequ)(SSDACH)$]^+$

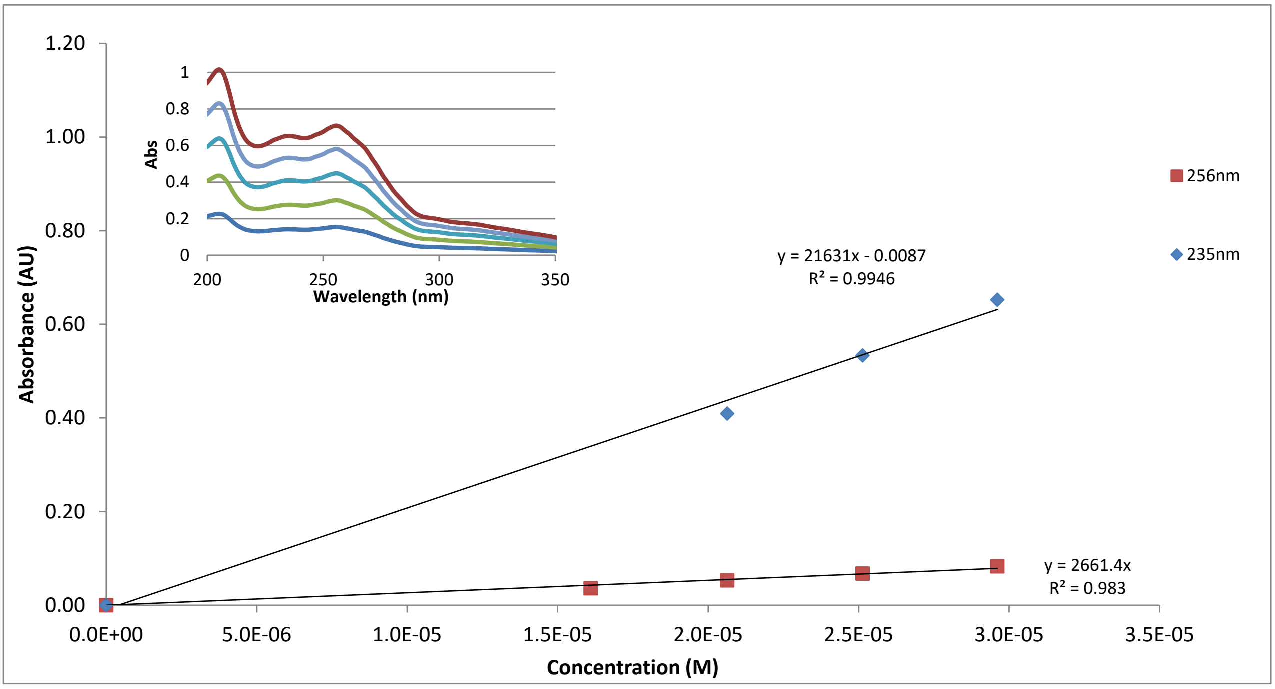

Figure S17: Titration of a stock solution of [Pt(DiBequ)(SSDACH)]$^+$ into a known concentration in H_2O and the resulting extinction coefficient calculated based on the two main peaks at 256 nm (red) and 235 nm (blue).

[Pt(DiBequ)(RRDACH)]⁺

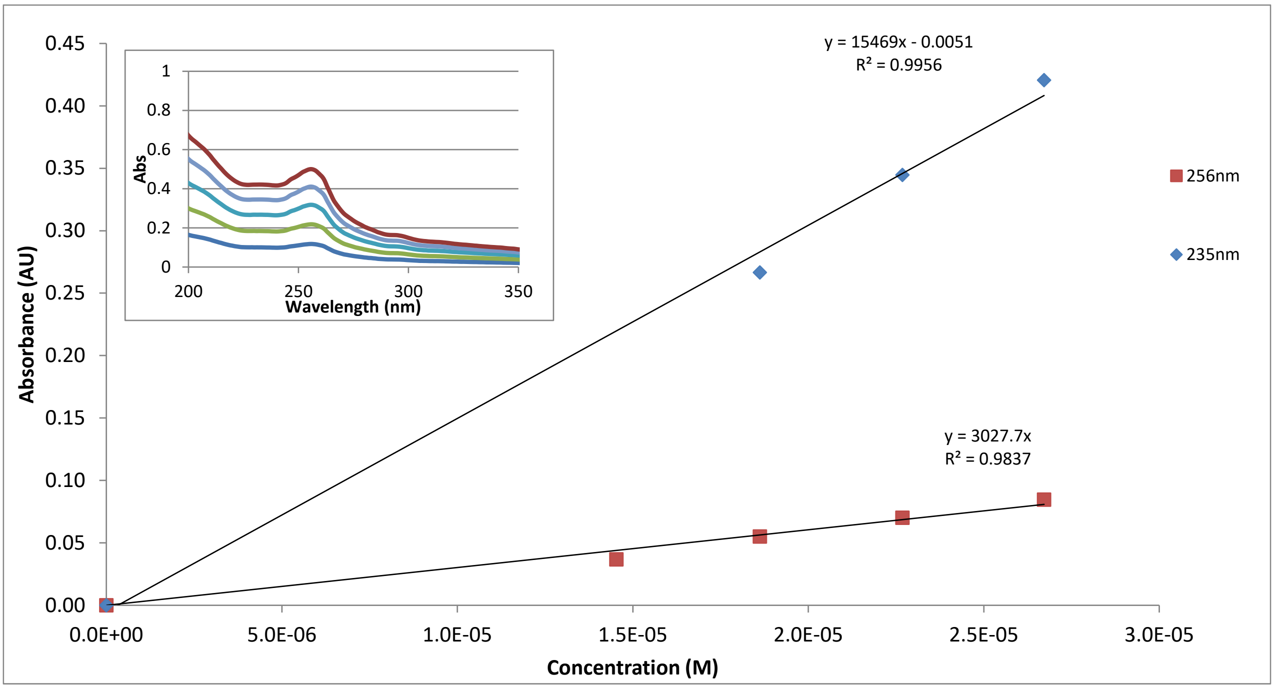

Figure S18: Titration of a stock solution of [Pt(Dibequ)(RRDACH)]⁺ into a known concentration in H_2O and the resulting extinction coefficient calculated based on the two main peaks at 256 nm (red) and 235 nm (blue).

UV Spectra

Ligands

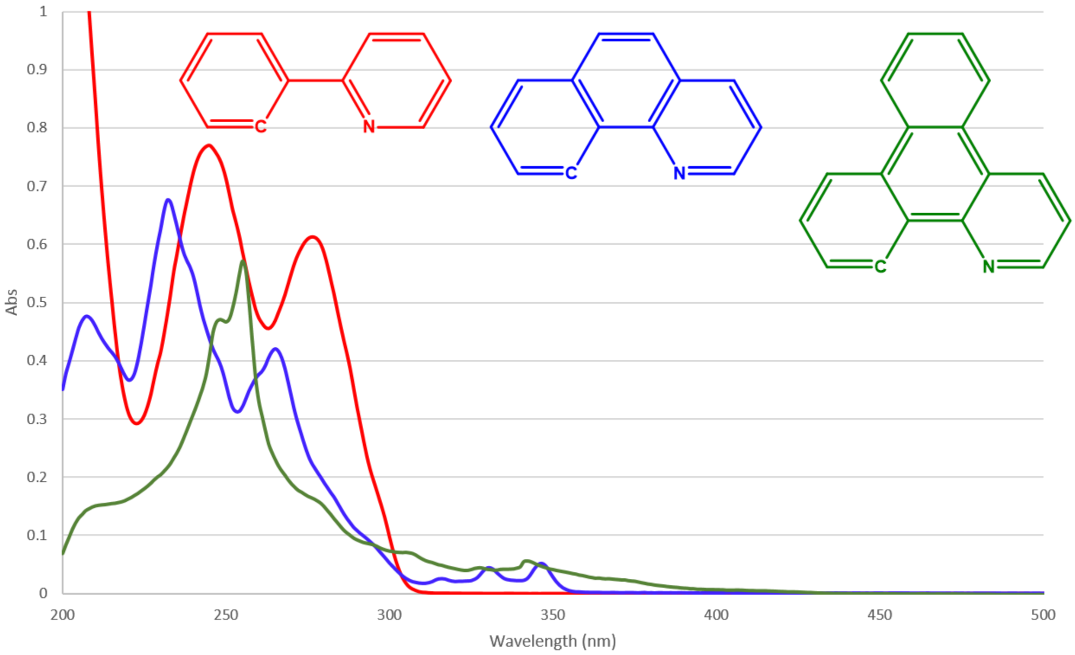

Figure S19: Absorbance of 2-phenylpyridine (red), Benzo[h]quinolone (blue) and dibenzo(f,h)quinolone (green) at the same concentration in ethanol and water solution.

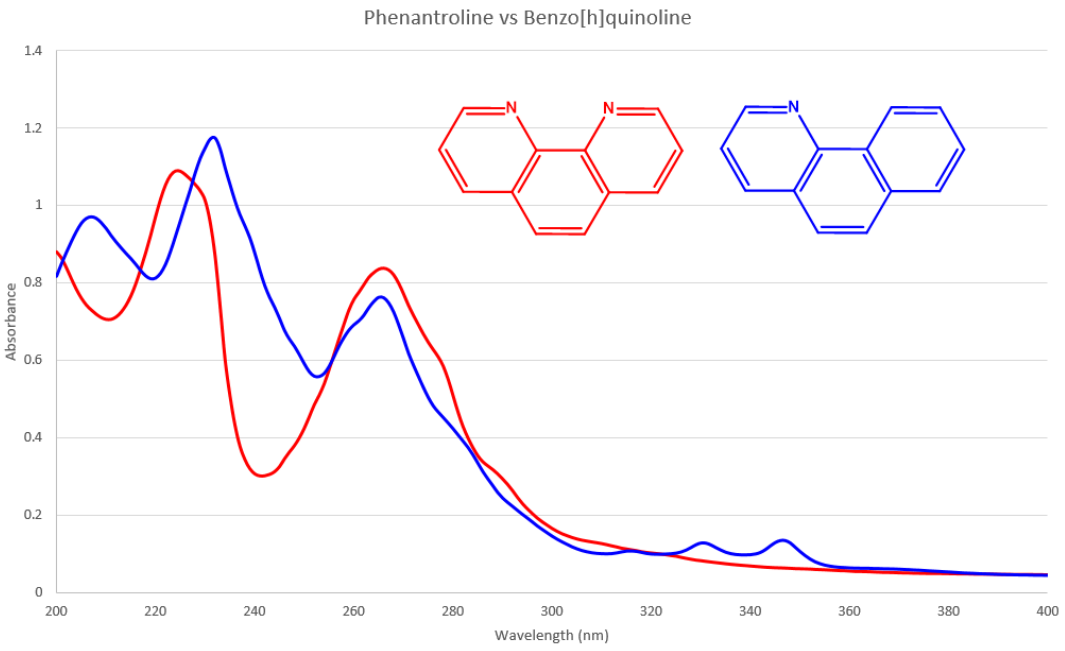

Figure S20: absorbance of phenathroline (red) and Benzo[h]quinolone (blue) at the same concentration in ethanol and water solution.

Complexes

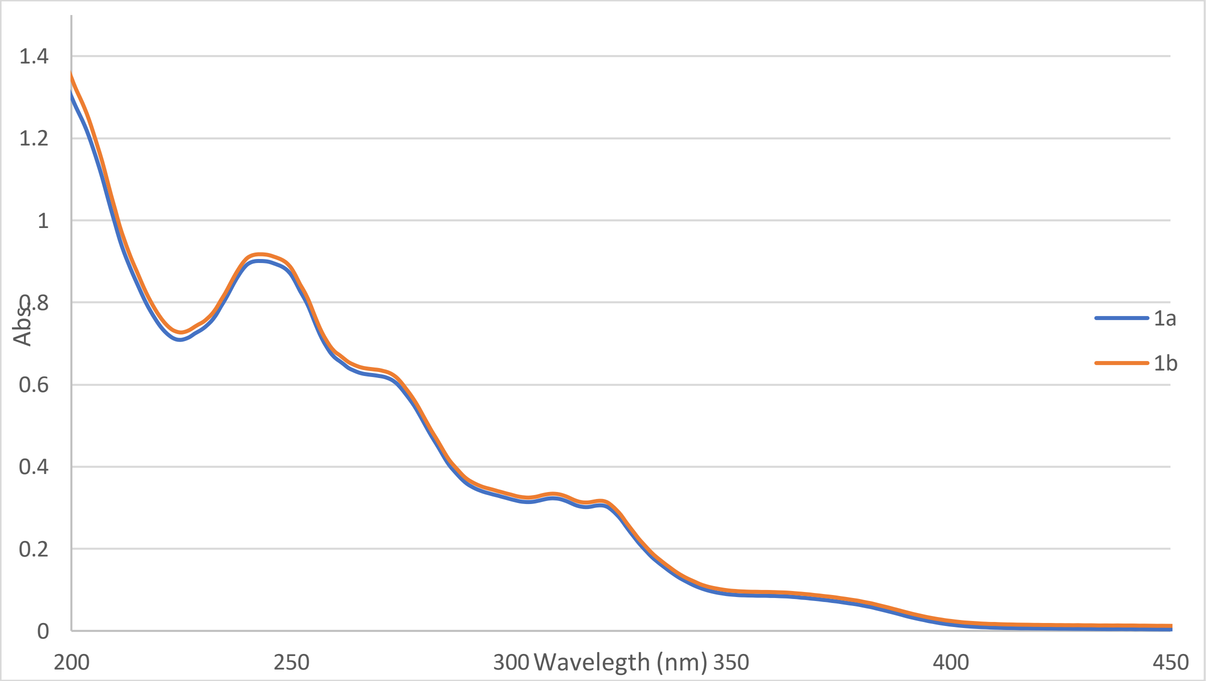

Figure S21: absorbance of **1a** (blue) and **1b** (orange) at the same concentration in water.

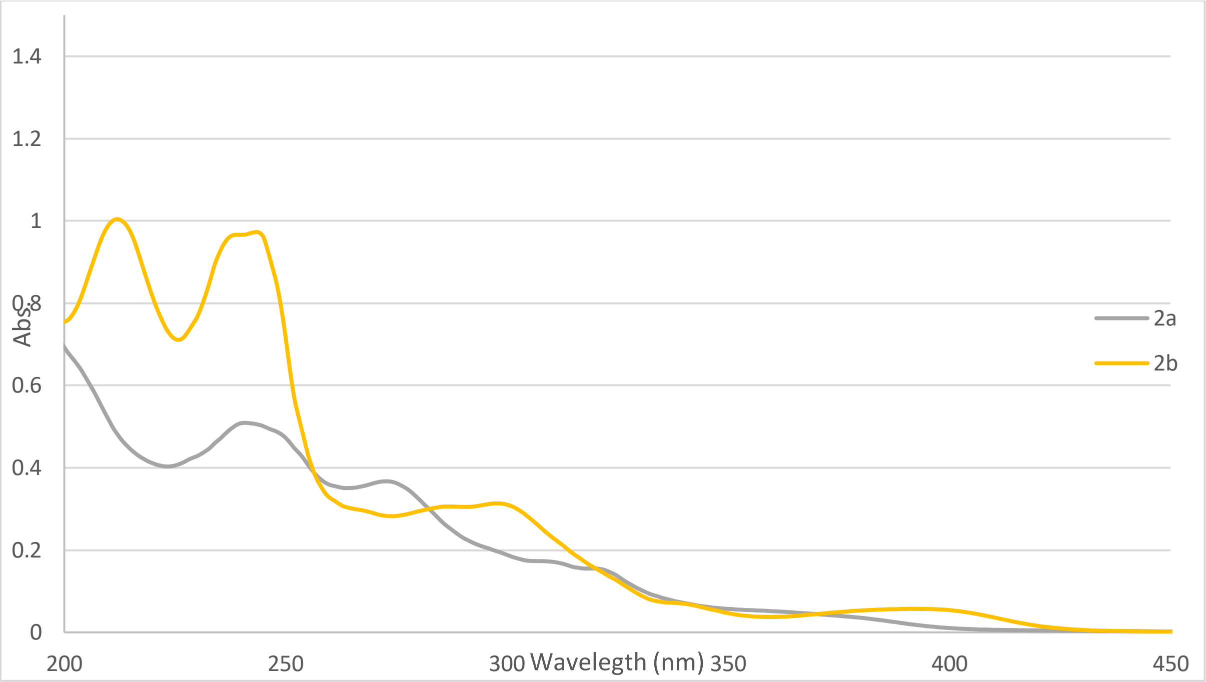

Figure S22: absorbance of **2a** (grey) and **2b** (yellow) at the same concentration in water.

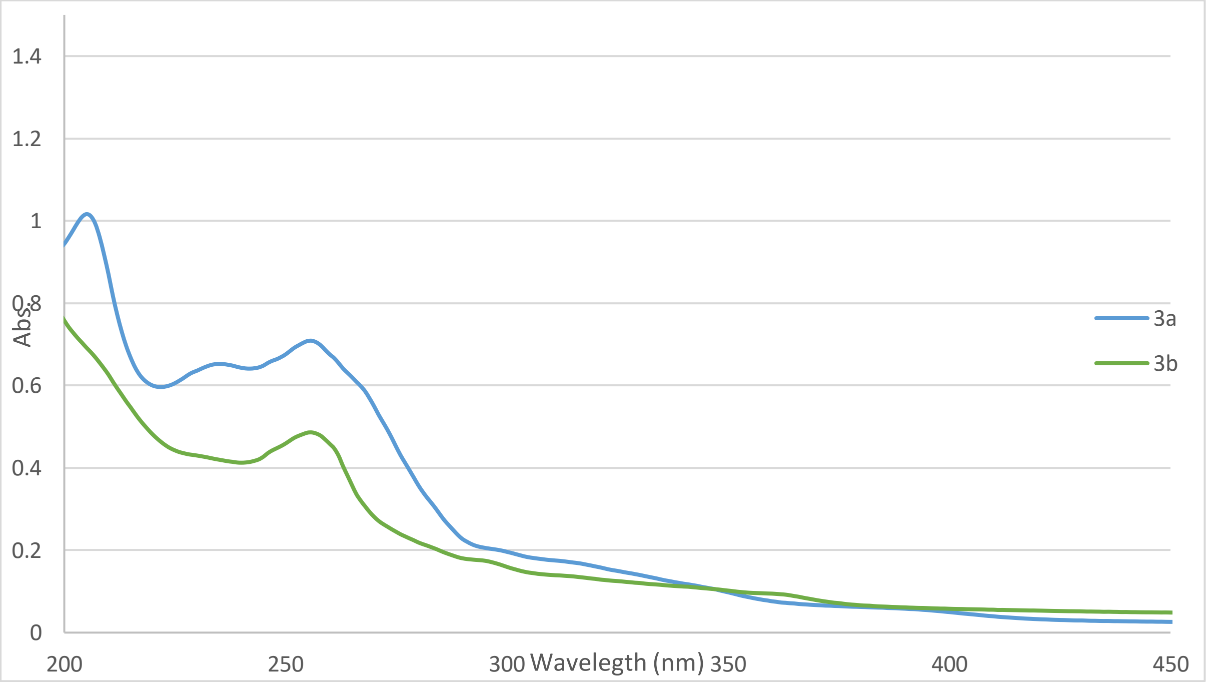

Figure S23: absorbance of **3a** (blue) and **3b** (green) at the same concentration in water.

Lipophilicity

Lipophilicity was calculated using RP-HPLC using a standard method whereby a stock solution was injected at different isocratic ratios ranging from 70-90 % solvent B (organic) at a flow rate of 1 $mLmin^{-1}$[1–3]. The subsequent peaks were recorded, and K was calculated as per equation S1.

Equation S1

$$k = \frac{t_r - t_0}{t_0}$$

Where k is the capacity factor, t_r is the retention time of the analyte and t_0 is the dead time.

For each compound the stock solution was run at 5 different isocratic ratios each repeated 3 times. Logk' was then calculated and plotted against the concentration of ACN in the mobile phase. The resulting linier equations could then be used to calculate log kw, expressed by equation S2.

Equation S2

$$\log k = S\varphi + \log k_w$$

Where S is the slope, φ is the concentration of ACN in the mobile phase and $\log k_w$ represents the capacity factor of the compound in 100% water.

Figure S1 illustrates the linear plots of logK' against % ACN including the resulting equation. These experiments were undertaken on an Agilent Technologies 1260 Infinity machine equipped with a Phenomenex Onyx™ Monolithic C18 reverse phase column (100 × 4.6 mm, 130 Å). The mobile phase comprised of 0.06% TFA in water (solvent A) and 0.06% TFA in ACN:H_2O (90 : 10, solvent B). The dead time was determined using potassium iodide as an external dead volume marker.

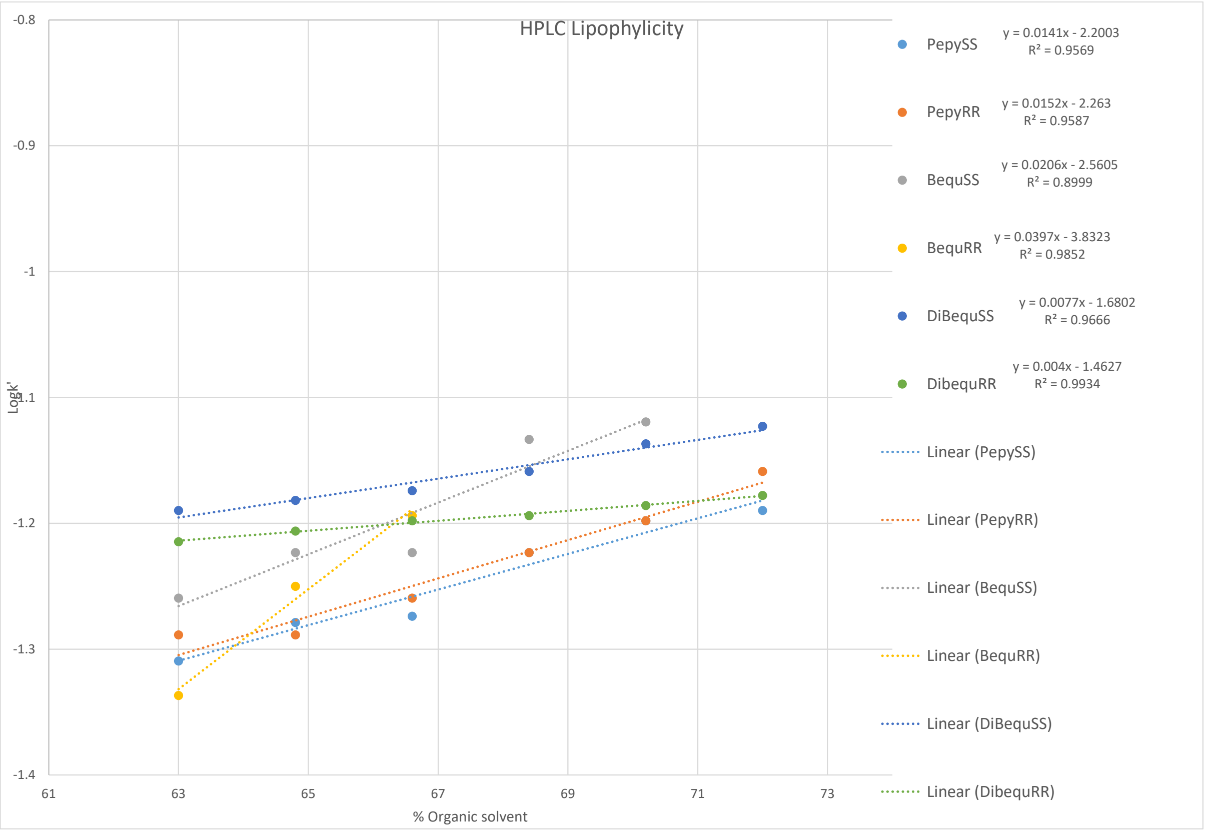

Figure S24: HPLC retention times for complexes **1-3** plotted against the organic solvent % present in the isocratic run.

CD

[Pt(Pepy)(DACH)]$^+$

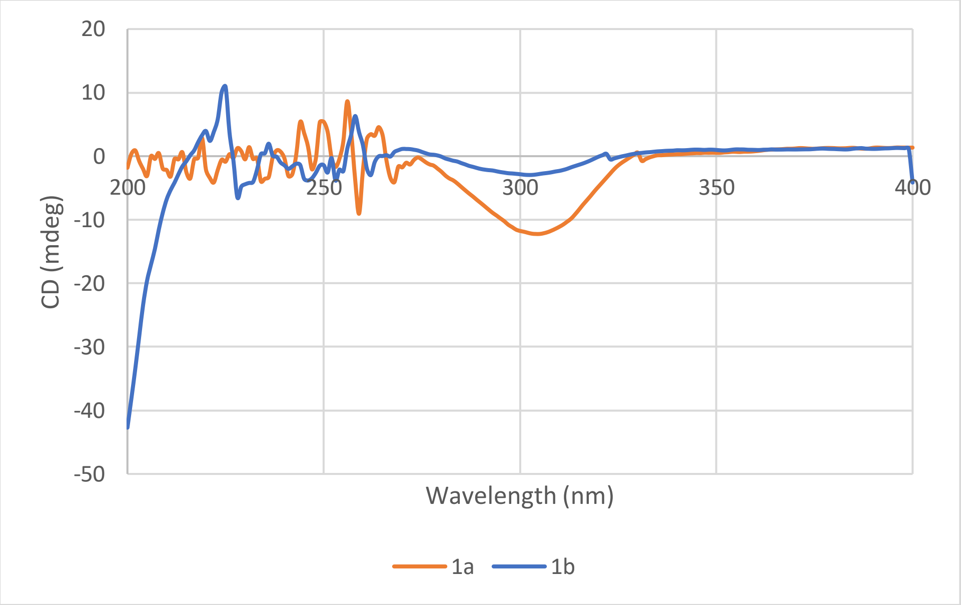

Figure S25: CD spectra of **1a** ([Pt(Pepy)(SSDACH)]$^+$) and **1b** ([Pt(Pepy)(SSDACH)]$^+$) at room temperature in the 200–400 nm range, using a 10 mm quartz cell, corrected for solvent baseline, measured in H_2O

[Pt(Bequ)(DACH)]$^+$

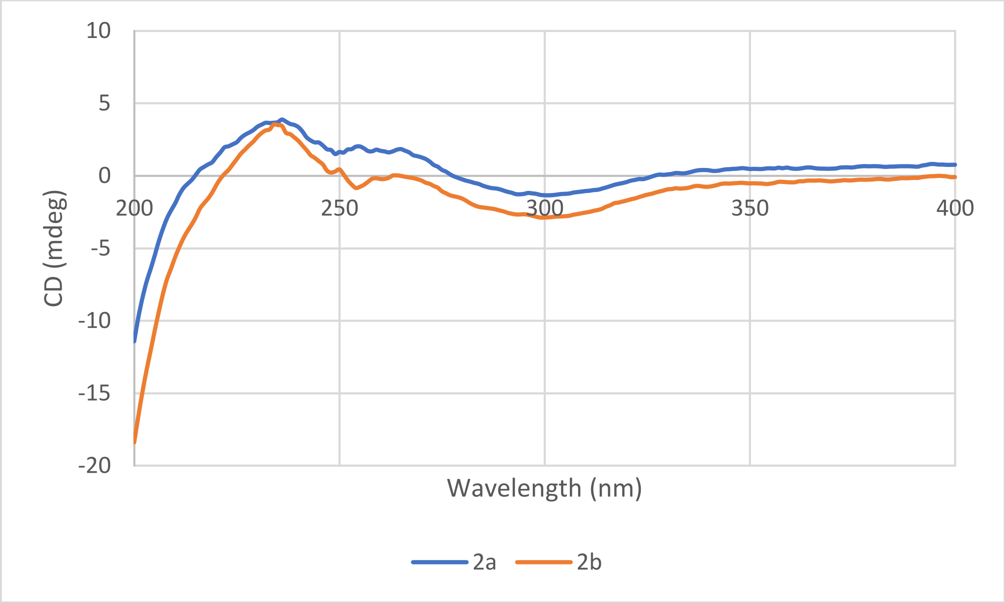

Figure S26: CD spectra of **2a** ([Pt(Bequ)(SSDACH)]$^+$) and **2b** ([Pt(Bequ)(SSDACH)]$^+$) at room temperature in the 200–400 nm range, using a 10 mm quartz cell, corrected for solvent baseline, measured in H_2O

[Pt(DiBequ)(DACH)]+

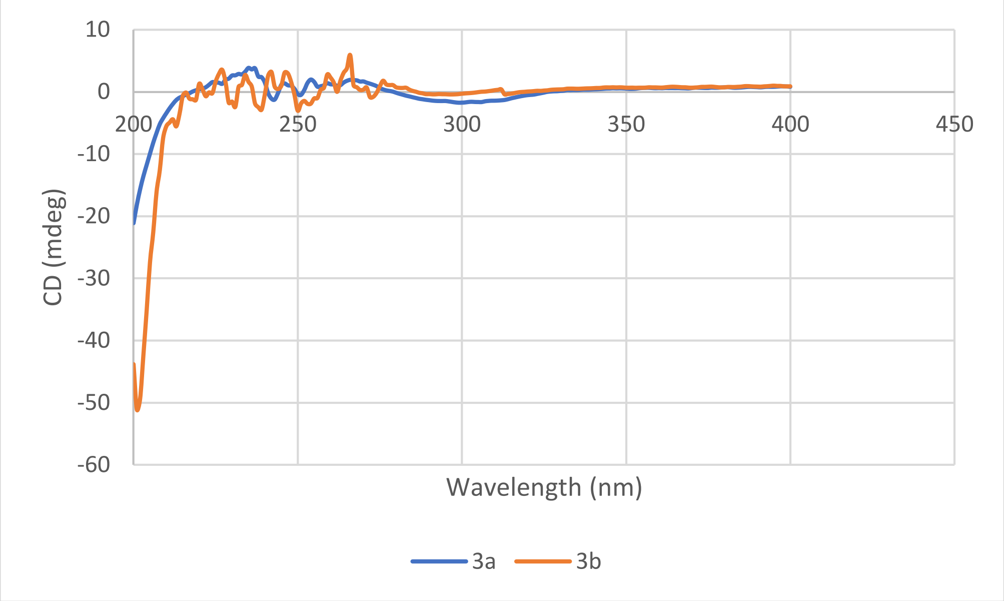

Figure S27: CD spectra of **3a** ([Pt(DiBequ)(SSDACH)]$^{+}$) and **3b** ([Pt(DiBequ)(SSDACH)]$^{+}$) at room temperature in the 200–400 nm range, using a 10 mm quartz cell, corrected for solvent baseline, measured in H_2O

HPLC

[Pt(Pepy)(SSDACH)]$^+$

```
Acq. Operator   : SYSTEM                         Seq. Line :    2
Acq. Instrument : LC1260                          Location : Vial 41
Injection Date  : 8/27/2021 10:42:12 AM                Inj :    1
                                                Inj Volume : 5.000 µl
Method          : D:\BRONDWYN\DATA\20210827_ACYCLODACHPT(IV) 2021-08-27 10-09-35\0 TO 100
                  OVER 15 MINS 5UL.M (Sequence Method)
Last changed    : 8/27/2021 10:09:36 AM by SYSTEM
```

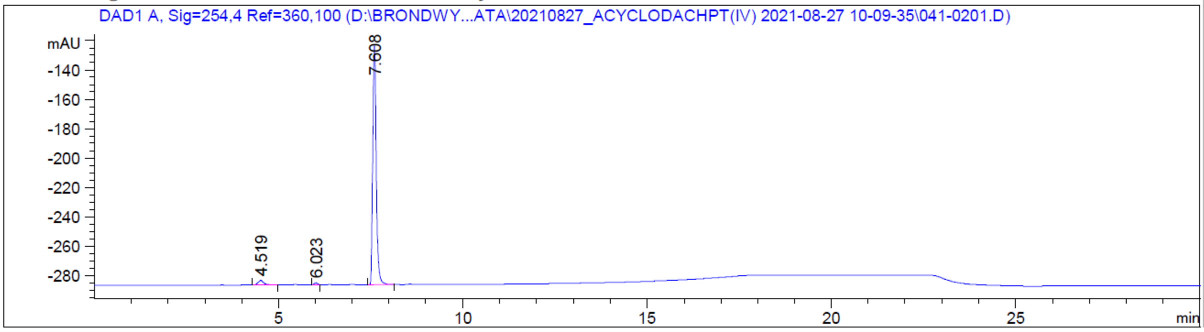

Figure S28: HPLC trace of **1a** measured on a Agilent Technologies 1260 Infinity machine equipped with a Phenomenex Onyx™ Monolithic C18 reverse phase column (100 × 4.6 mm, 130 Å). Sample solutions were made up in H_2O and injected at a 0-100 gradient A to B over 15 minutes with a 15 minute flush in-between samples.

[Pt(Pepy)(RRDACH)]$^+$

```
Acq. Operator   : SYSTEM                         Seq. Line :    2
Acq. Instrument : LC1260                          Location : Vial 42
Injection Date  : 6/15/2021 2:59:53 PM                 Inj :    1
                                                Inj Volume : 10.000 µl
Method          : D:\BRONDWYN\DATA\06152021_BM 2021-06-15 14-27-05\0 TO 100 OVER 15 MINS 10UL
                  .M (Sequence Method)
Last changed    : 6/15/2021 2:27:05 PM by SYSTEM
```

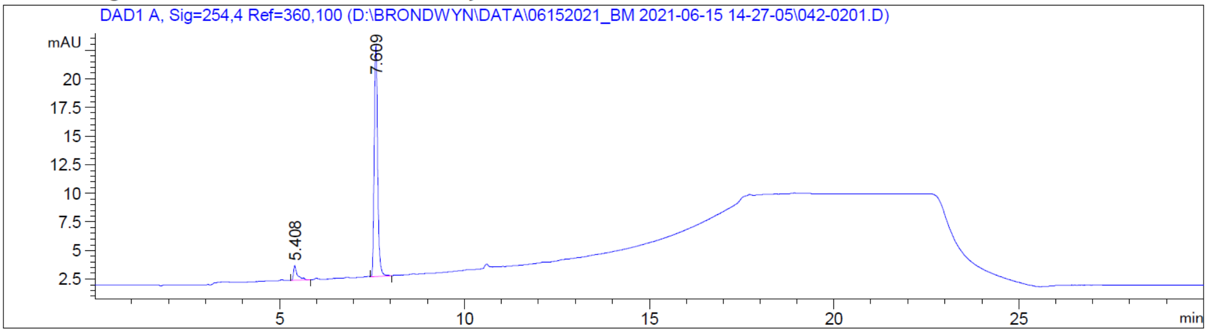

Figure S29: HPLC trace of **1b** measured on a Agilent Technologies 1260 Infinity machine equipped with a Phenomenex Onyx™ Monolithic C18 reverse phase column (100 × 4.6 mm, 130 Å). Sample solutions were made up in H_2O and injected at a 0-100 gradient A to B over 15 minutes with a 15 minute flush in-between samples.

[Pt(Bequ)(SSDACH)]+

Acq. Operator : SYSTEM Seq. Line : 3
Acq. Instrument : LC1260 Location : Vial 43
Injection Date : 6/15/2021 3:31:18 PM Inj : 1
Inj Volume : 10.000 µl
Method : D:\BRONDWYN\DATA\06152021_BM 2021-06-15 14-27-05\0 TO 100 OVER 15 MINS 10UL .M (Sequence Method)
Last changed : 6/15/2021 2:27:05 PM by SYSTEM

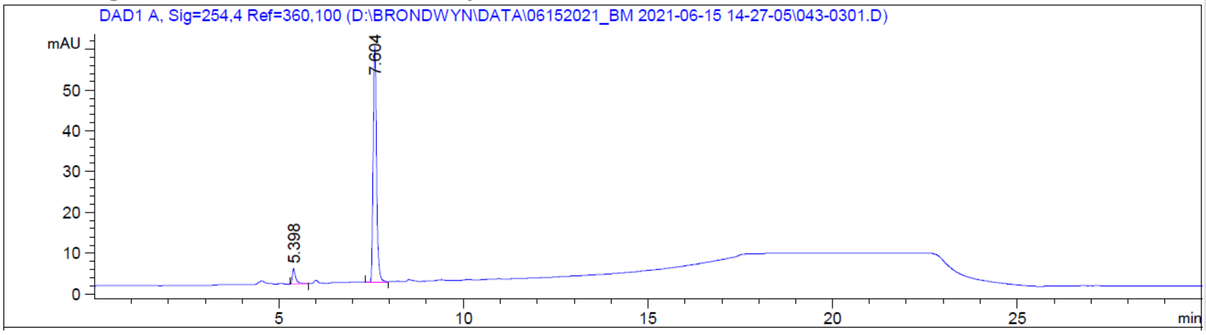

Figure S30: HPLC trace of **2a** measured on a Agilent Technologies 1260 Infinity machine equipped with a Phenomenex Onyx™ Monolithic C18 reverse phase column (100 × 4.6 mm, 130 Å). Sample solutions were made up in H_2O and injected at a 0-100 gradient A to B over 15 minutes with a 15 minute flush in-between samples.

[Pt(Bequ)(RRDACH)]+

Acq. Operator : SYSTEM Seq. Line : 4
Acq. Instrument : LC1260 Location : Vial 44
Injection Date : 6/15/2021 4:02:44 PM Inj : 1
Inj Volume : 10.000 µl
Method : D:\BRONDWYN\DATA\06152021_BM 2021-06-15 14-27-05\0 TO 100 OVER 15 MINS 10UL .M (Sequence Method)
Last changed : 6/15/2021 2:27:05 PM by SYSTEM

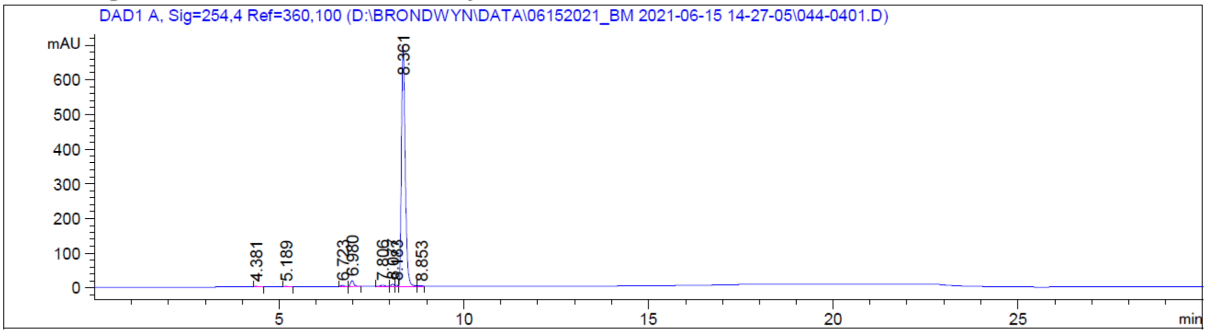

Figure S31: HPLC trace of **2b** measured on a Agilent Technologies 1260 Infinity machine equipped with a Phenomenex Onyx™ Monolithic C18 reverse phase column (100 × 4.6 mm, 130 Å). Sample solutions were made up in H_2O and injected at a 0-100 gradient A to B over 15 minutes with a 15 minute flush in-between samples.

[Pt(DiBequ)(SSDACH)]+

Acq. Operator : SYSTEM
Acq. Instrument : LC1260
Injection Date : 6/21/2021 1:02:01 PM
Seq. Line : 5
Location : Vial 24
Inj : 1
Inj Volume : 10.000 µl
Method : D:\BRONDWYN\DATA\21_05_31_CYCLODACH 2021-06-21 10-55-10\0 TO 100 OVER 15 MINS 10UL_EDT.M (Sequence Method)
Last changed : 6/21/2021 10:55:10 AM by SYSTEM

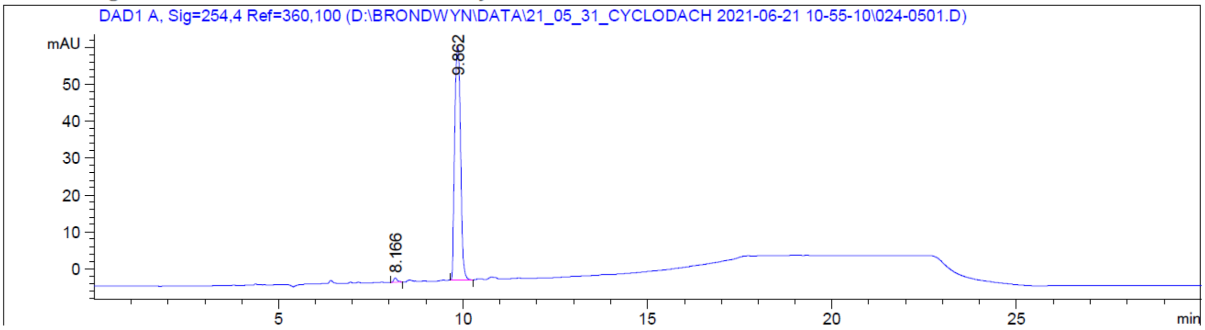

Figure S32: HPLC trace of **3a** measured on a Agilent Technologies 1260 Infinity machine equipped with a Phenomenex Onyx™ Monolithic C18 reverse phase column (100 × 4.6 mm, 130 Å). Sample solutions were made up in H_2O and injected at a 0-100 gradient A to B over 15 minutes with a 15 minute flush in-between samples.

[Pt(DiBequ)(RRDACH)]+

Acq. Operator : SYSTEM
Acq. Instrument : LC1260
Injection Date : 2/9/2021 3:21:55 PM
Seq. Line : 4
Location : Vial 41
Inj : 1
Inj Volume : 10.000 µl
Method : D:\BRONDWYN\DATA\20210209_BM 2021-02-09 13-46-22\0 TO 100 OVER 15 MINS 10UL .M (Sequence Method)
Last changed : 2/9/2021 1:46:22 PM by SYSTEM

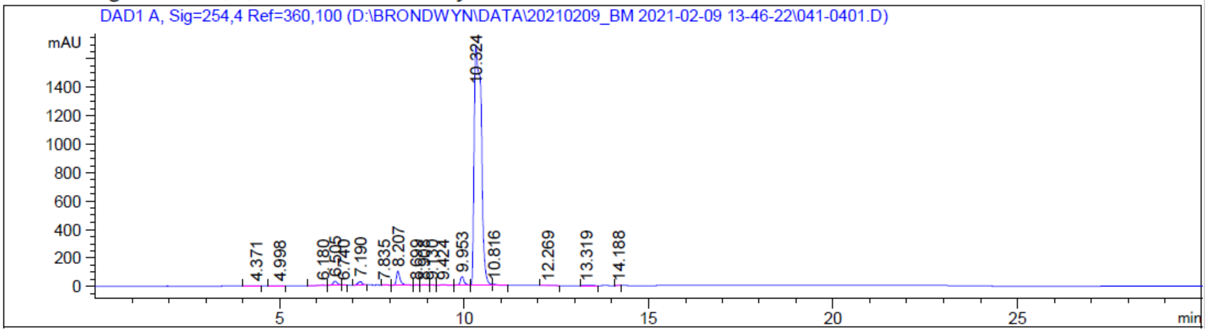

Figure S33: HPLC trace of **3b** measured on a Agilent Technologies 1260 Infinity machine equipped with a Phenomenex Onyx™ Monolithic C18 reverse phase column (100 × 4.6 mm, 130 Å). Sample solutions were made up in H_2O and injected at a 0-100 gradient A to B over 15 minutes with a 15 minute flush in-between samples.

ESI-MS

[Pt(Pepy)(SSDACH)]$^+$

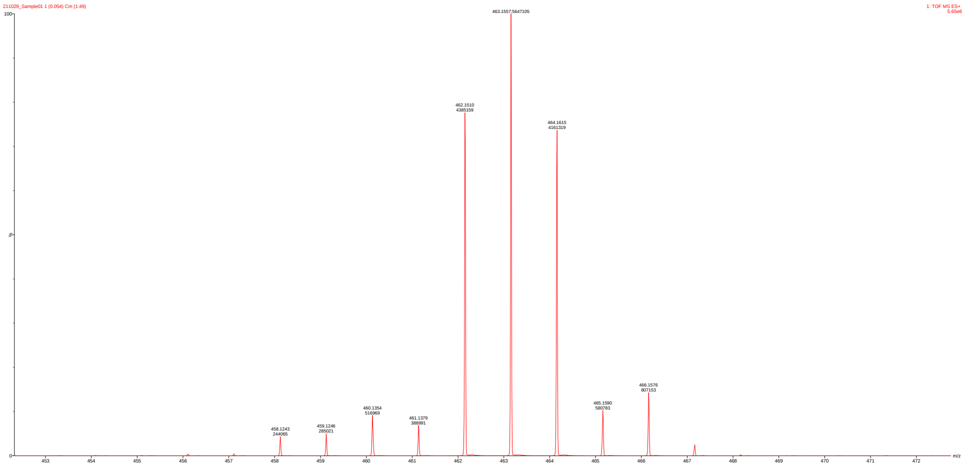

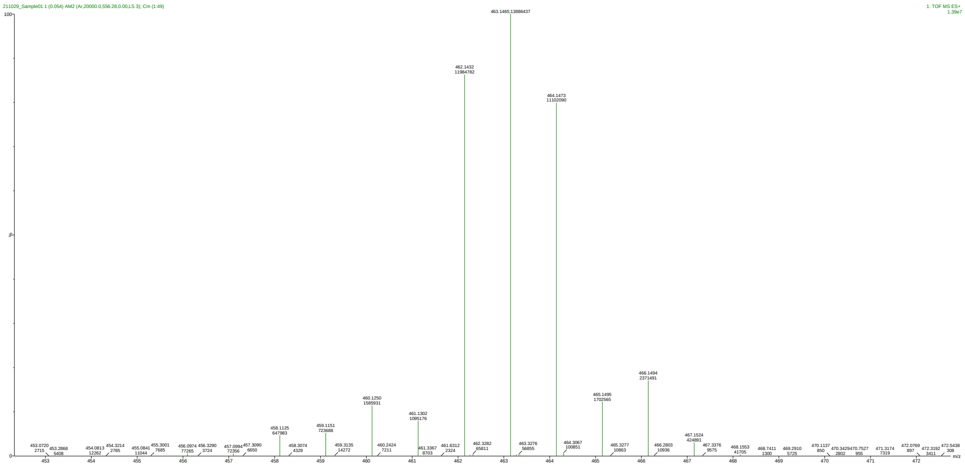

Figure S34: ESIMS spectrum of **1a** measured on a Waters TQ-MS triple quadrupole mass spectrometer. Sample solutions were made up to 0.5 mM in H_2O and flowed at 0.1 mL/min.

[Pt(Pepy)(RRDACH)]$^+$

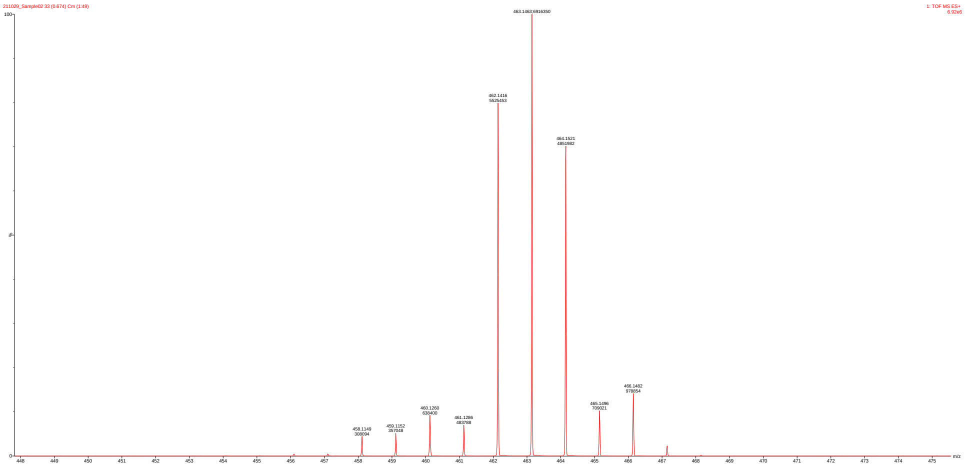

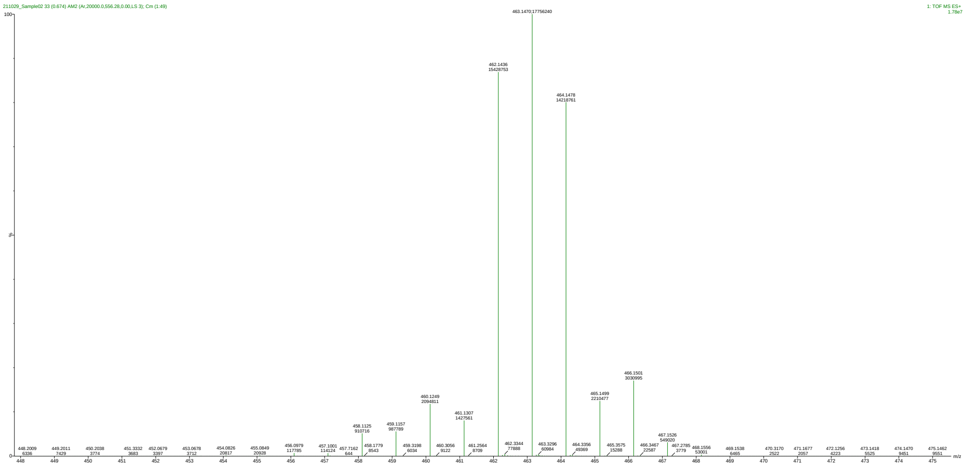

Figure S35: ESIMS spectrum of **1b** measured on a Waters TQ-MS triple quadrupole mass spectrometer. Sample solutions were made up to 0.5 mM in H_2O and flowed at 0.1 mL/min.

$[Pt(Bequ)(SSDACH)]^+$

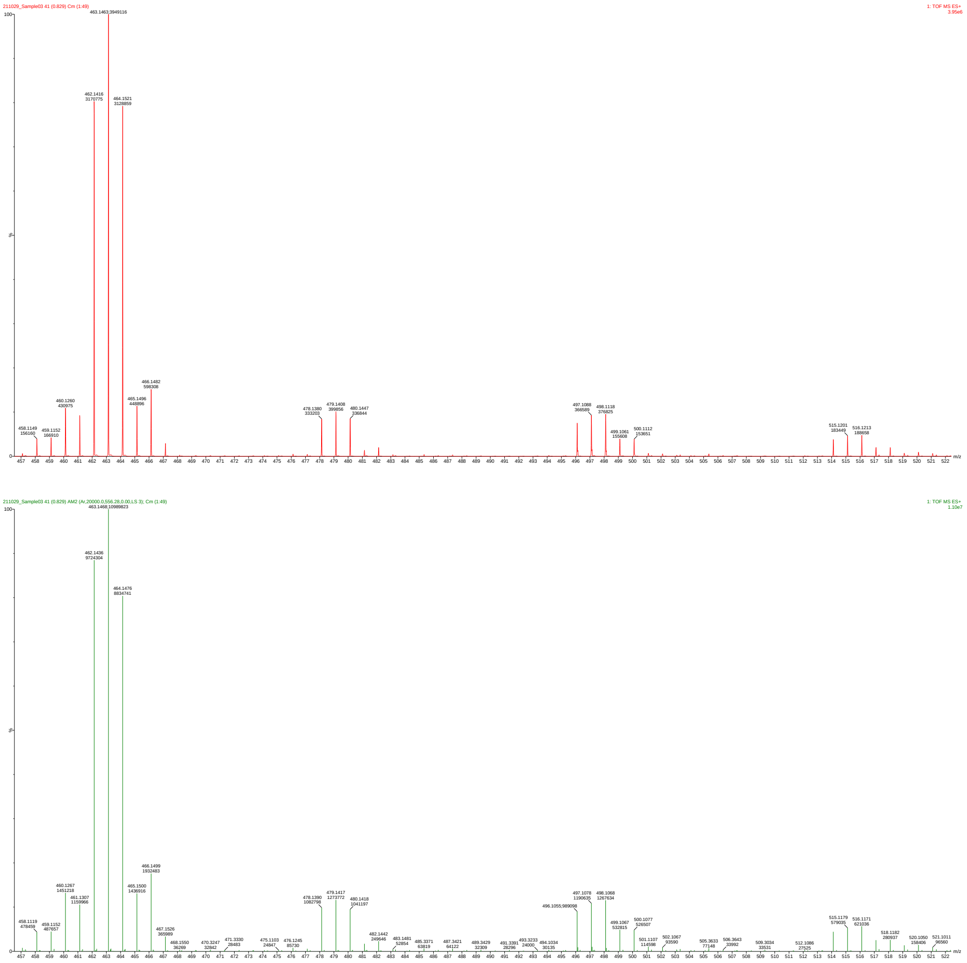

Figure S36: ESIMS spectrum of **2a** measured on a Waters TQ-MS triple quadrupole mass spectrometer. Sample solutions were made up to 0.5 mM in H_2O and flowed at 0.1 mL/min.

[Pt(Bequ)(RRDACH)]$^{+}$

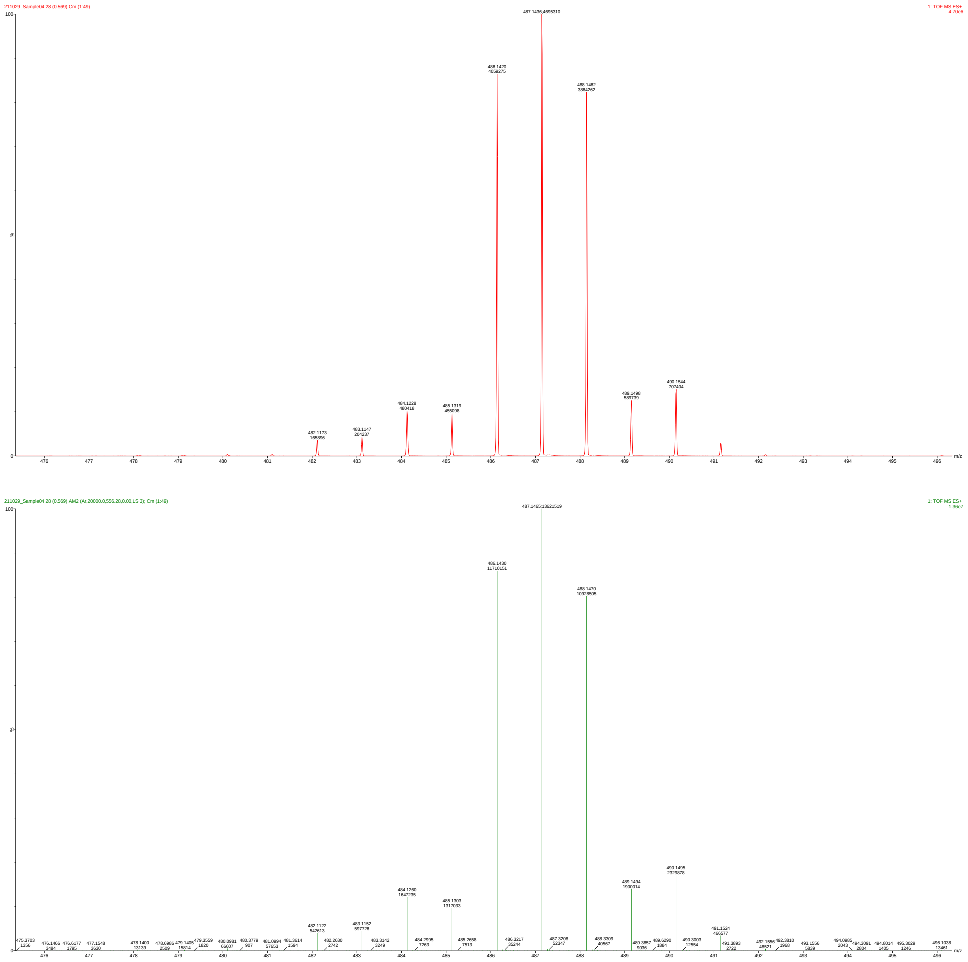

Figure S37: ESIMS spectrum of **2b** measured on a Waters TQ-MS triple quadrupole mass spectrometer. Sample solutions were made up to 0.5 mM in H_2O and flowed at 0.1 mL/min.

[Pt(DiBequ)(SSDACH)$]^{+}$

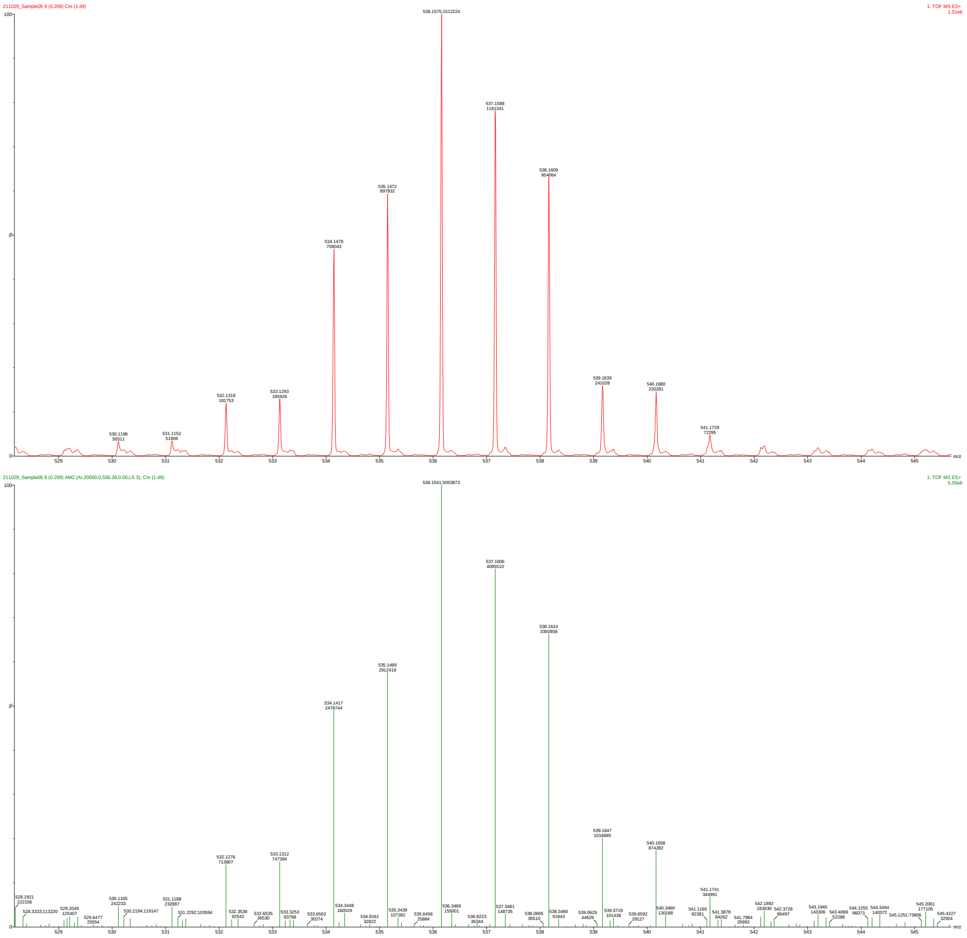

Figure S38: ESIMS spectrum of **3a** measured on a Waters TQ-MS triple quadrupole mass spectrometer. Sample solutions were made up to 0.5 mM in H_2O and flowed at 0.1 mL/min.

$[Pt(DiBequ)(RRDACH)]^+$

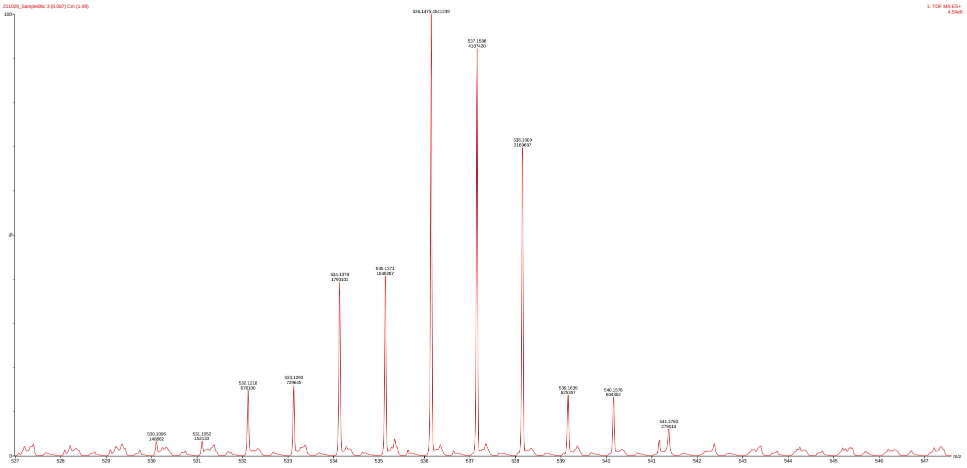

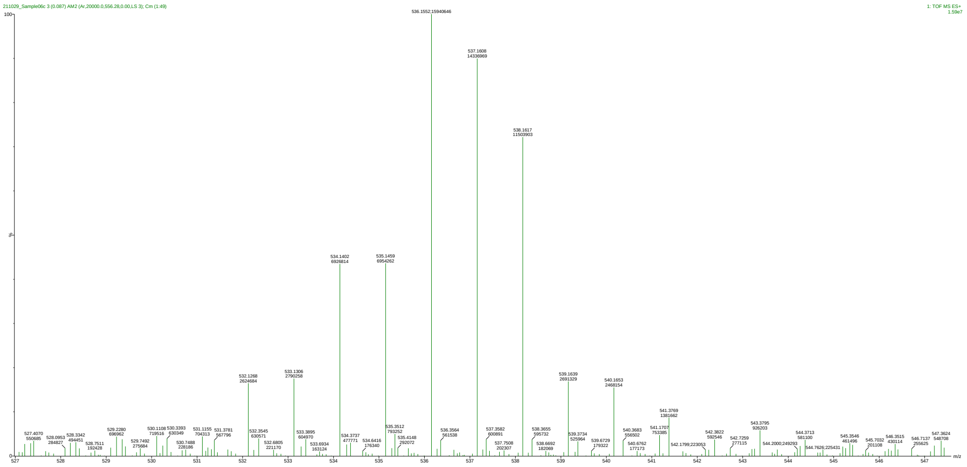

Figure S39: ESIMS spectrum of **3b** measured on a Waters TQ-MS triple quadrupole mass spectrometer. Sample solutions were made up to 0.5 mM in H_2O and flowed at 0.1 mL/min.

Fluorescence

QY

Quantum yields were obtained by following the IUPAC technical report on standards for photoluminescence quantum yield measurements in solution.[1] $Ru(bpy)_3$ was chosen as the best standard as per these guidelines and the quantum yield of the 9 complexes was calculated using equation S3 following the steps from the technical report.

Equation S3

$$\Phi_{st} = \Phi_s \frac{slope_{st} n_{st}^2}{slope_s\, n_s^2}$$

Where Φ_{st} and Φ_s is the quantum yield of the standard and the sample respectfully, n_{st} and n_s are the refractive indices of the solvents that the standard and sample are dissolved in respectively, and $slope_{st}$ and $slope_s$ are the slope of the line obtained from the plot of the area of florescence vs. absorbance of the standard and the sample respectfully.

DNA binding

UV

[Pt(Pepy)(SSDACH)]⁺

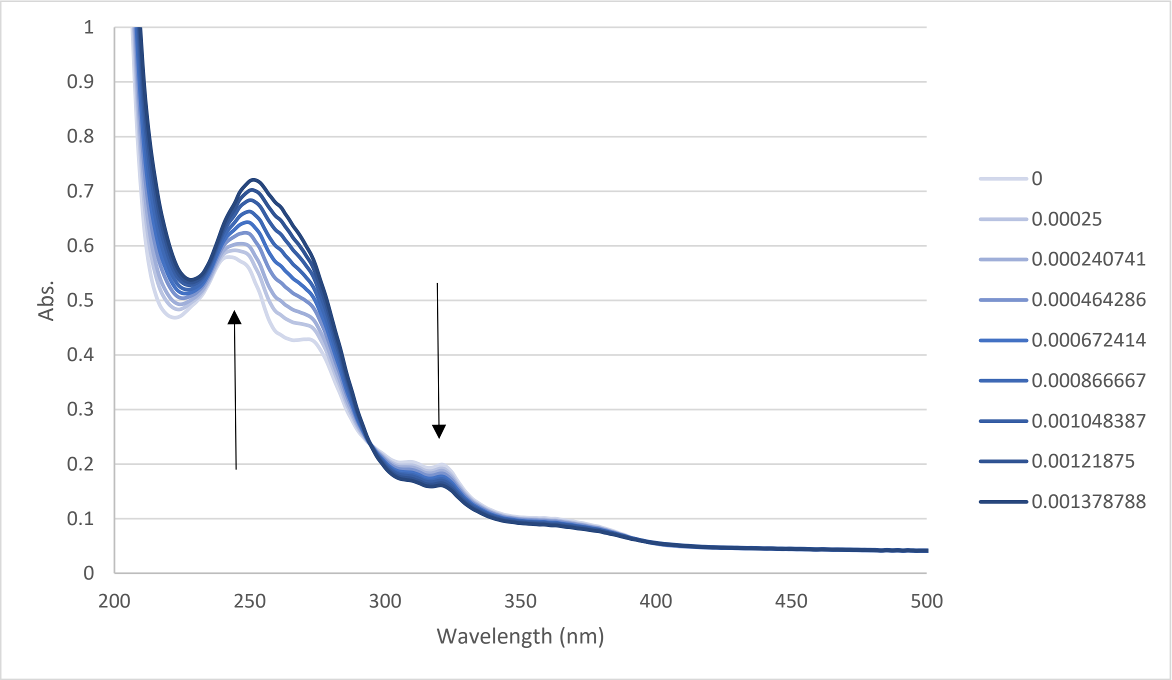

Figure S40: absorbance of **1a** with increasing concentration of ctDNA in Buffer. Up arrow indicates where absorbance is increasing upon the addition of ctDNA and down arrow indicates where absorbance is decreasing upon the addition of ctDNA.

[Pt(Pepy)(RRDACH)]+

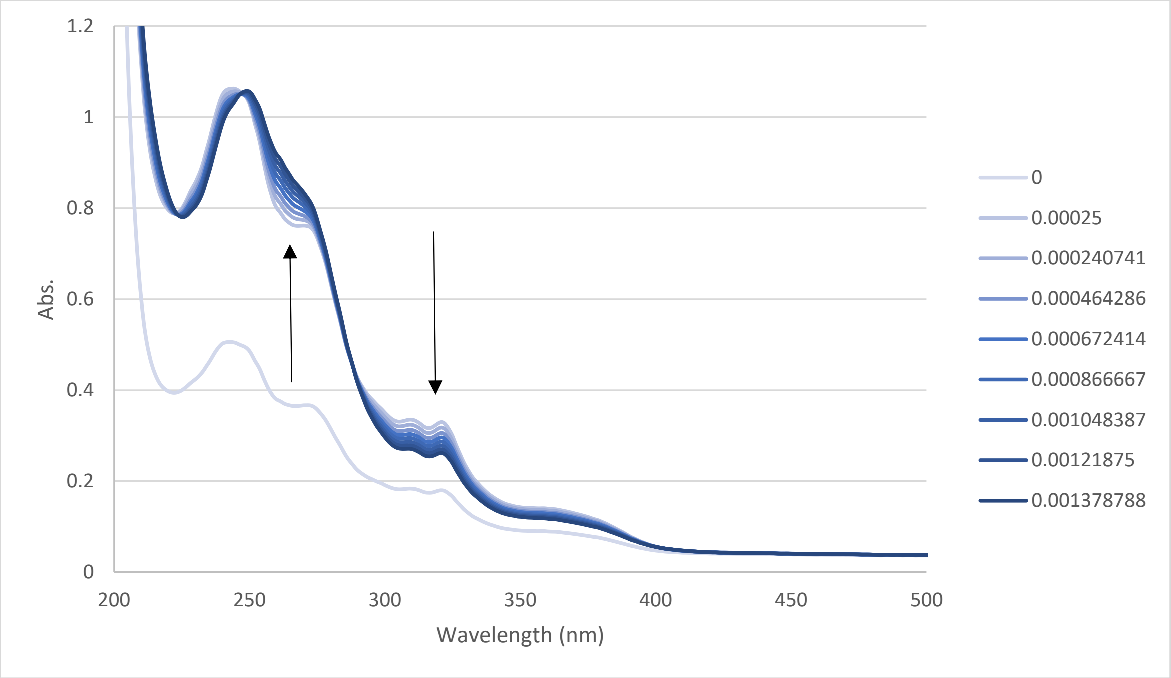

Figure S41: absorbance of **1b** with increasing concentration of ctDNA in Buffer. Up arrow indicates where absorbance is increasing upon the addition of ctDNA and down arrow indicates where absorbance is decreasing upon the addition of ctDNA.

[Pt(Bequ)(SSDACH)]+

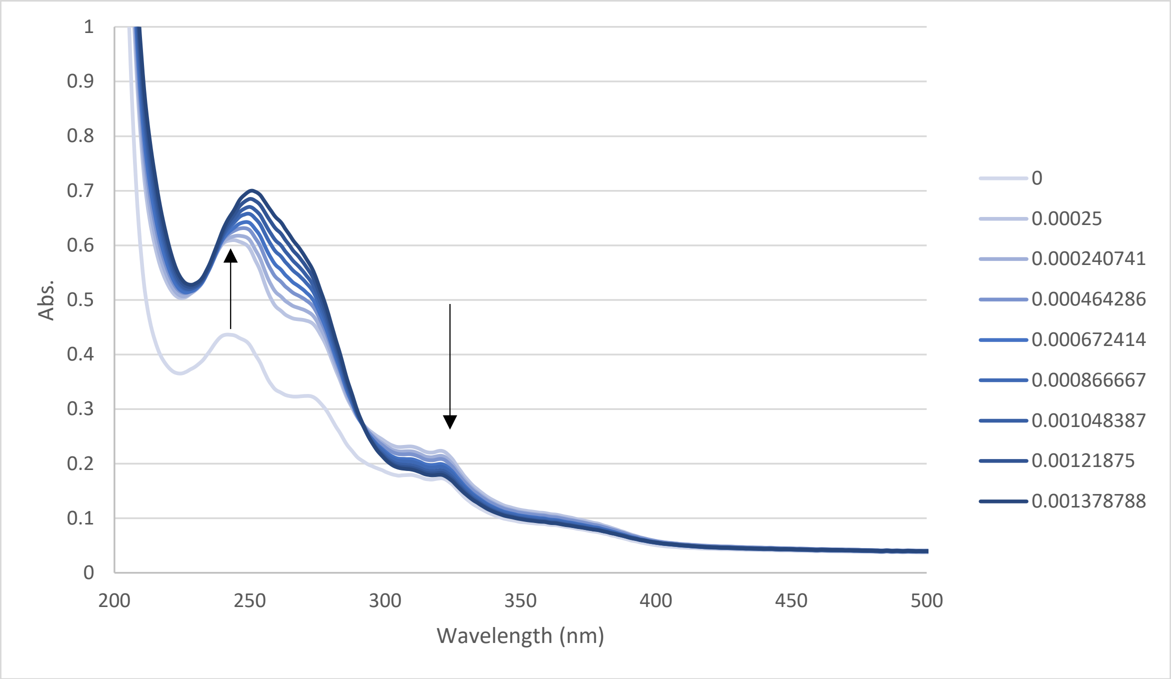

Figure S42: absorbance of **2a** with increasing concentration of ctDNA in Buffer. Up arrow indicates where absorbance is increasing upon the addition of ctDNA and down arrow indicates where absorbance is decreasing upon the addition of ctDNA.

[Pt(Bequ)(RRDACH)]⁺

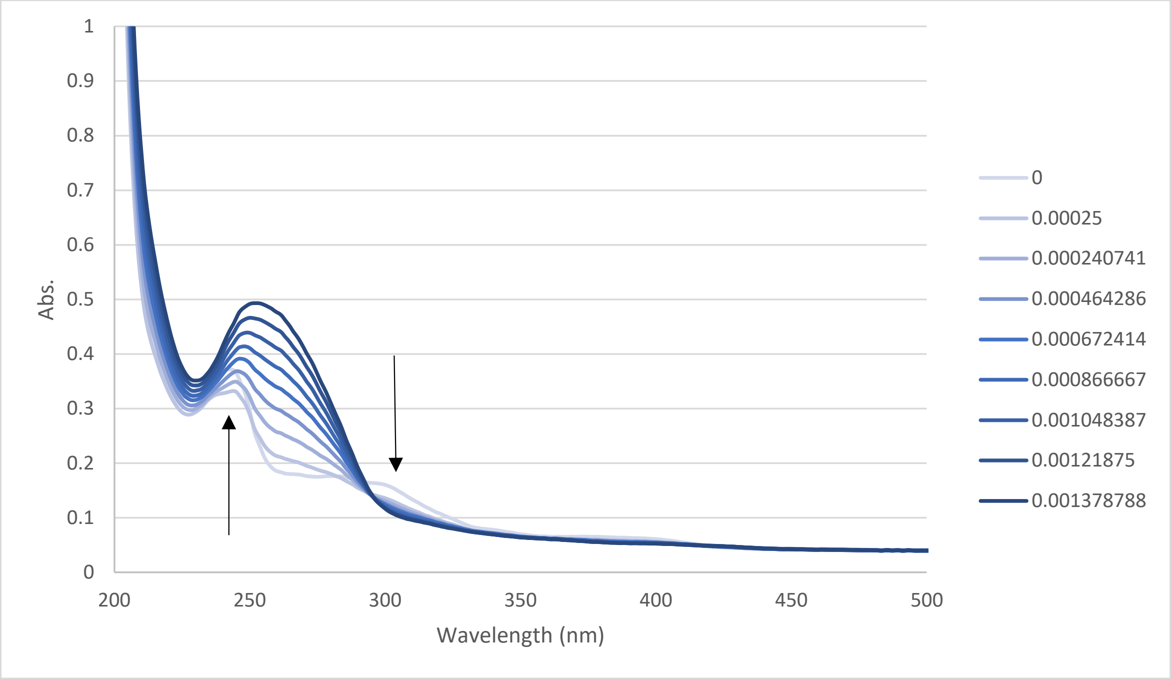

Figure S43: absorbance of **2b** with increasing concentration of ctDNA in Buffer. Up arrow indicates where absorbance is increasing upon the addition of ctDNA and down arrow indicates where absorbance is decreasing upon the addition of ctDNA.

[Pt(DiBequ)(SSDACH)]⁺

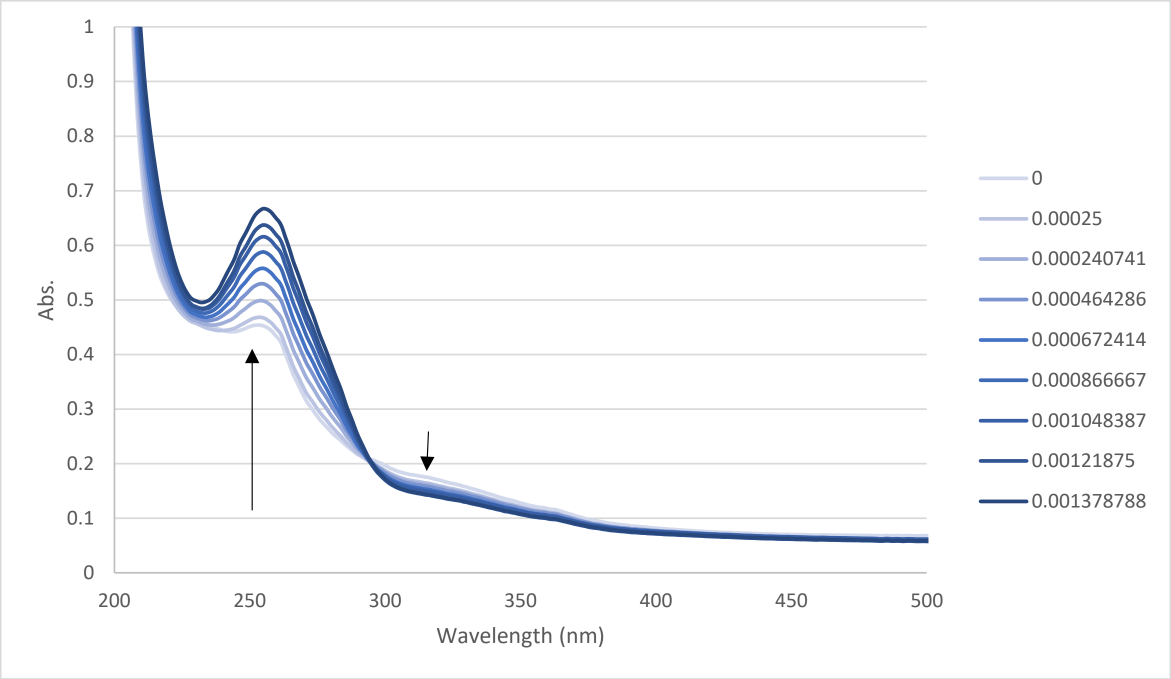

Figure S44: absorbance of **3a** with increasing concentration of ctDNA in Buffer. Up arrow indicates where absorbance is increasing upon the addition of ctDNA and down arrow indicates where absorbance is decreasing upon the addition of ctDNA.

[Pt(DiBequ)(RRDACH)]+

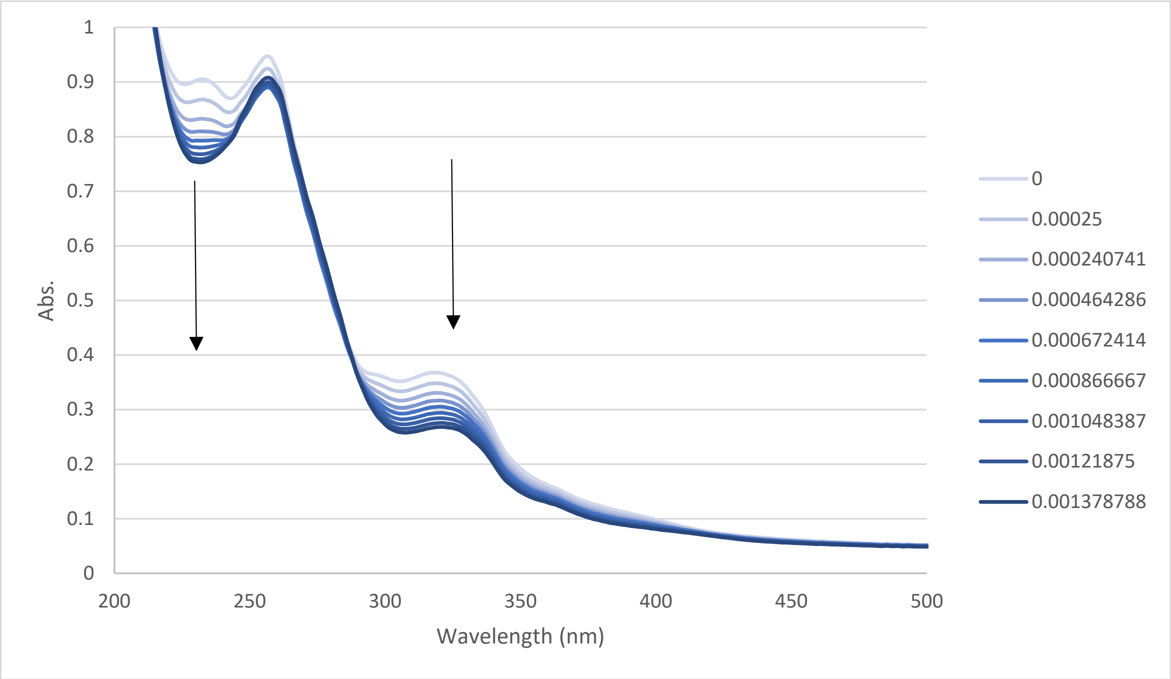

Figure S45: absorbance of **3b** with increasing concentration of ctDNA in Buffer. Up arrow indicates where absorbance is increasing upon the addition of ctDNA.

Inherent Fluorescence

[Pt(Pepy)(SSDACH)]+

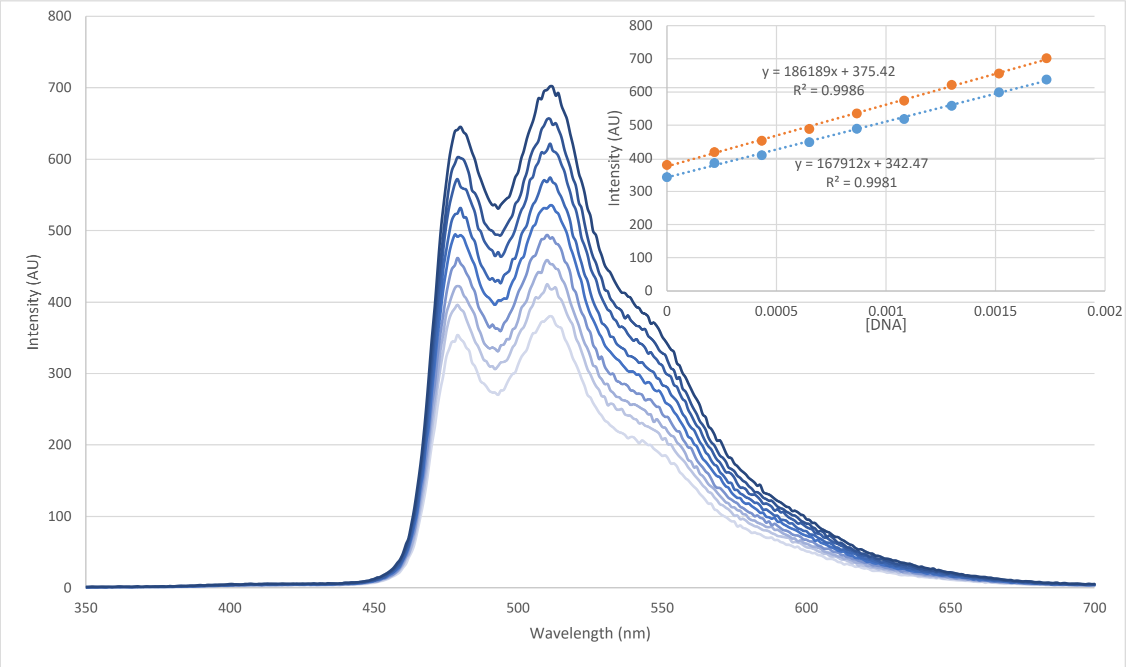

Figure S46: fluorescent intensity of **1a** with increasing concentration of ctDNA in Buffer (blue getting darker as DNA concentration increases). Insert shows graph of fluorescence intensification as concentration of DNA increases at 481 nm (blue) and 511 nm (orange).

[Pt(Pepy)(RRDACH)]$^+$

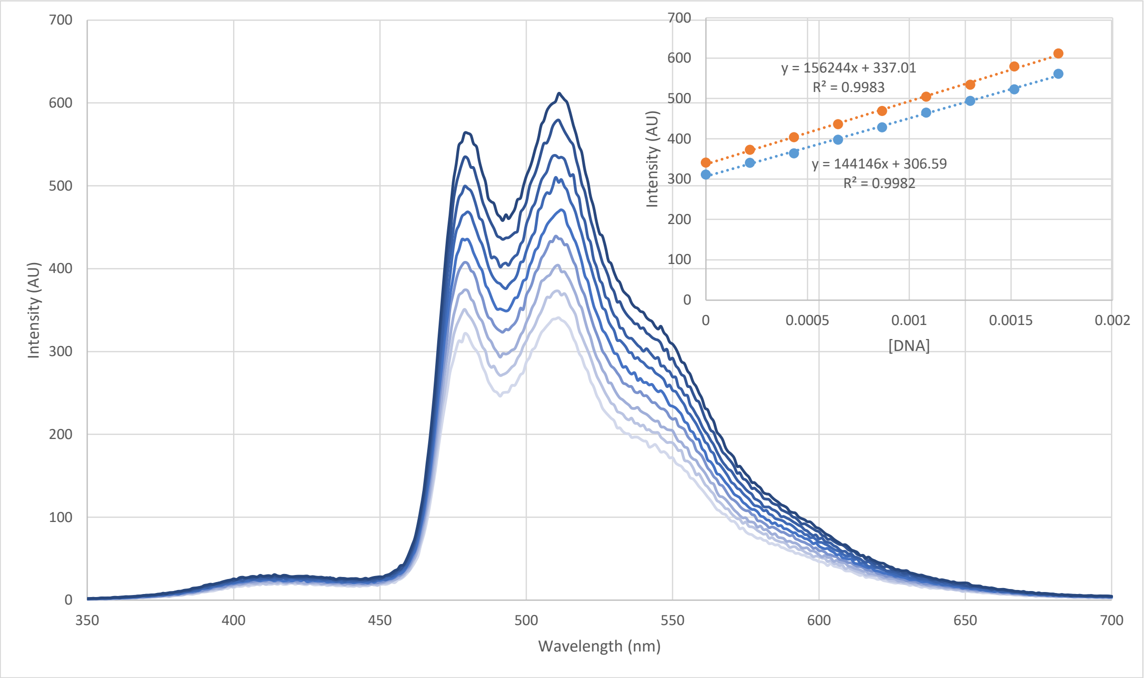

Figure S47: fluorescent intensity of **1b** with increasing concentration of ctDNA in Buffer (blue getting darker as DNA concentration increases). Insert shows graph of fluorescence intensification as concentration of DNA increases at 481 nm (blue) and 511 nm (orange).

[Pt(Bequ)(SSDACH)]$^+$

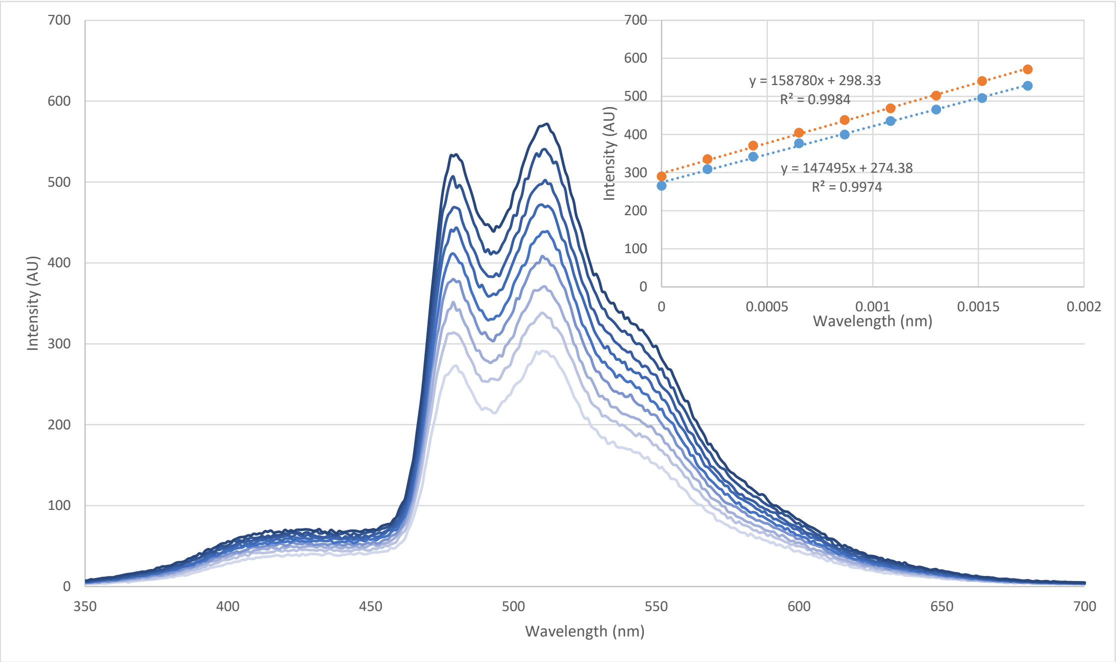

Figure S48: fluorescent intensity of **2a** with increasing concentration of ctDNA in Buffer (blue getting darker as DNA concentration increases). Insert shows graph of fluorescence intensification as concentration of DNA increases at 481 nm (blue) and 511 nm (orange).

$[Pt(Bequ)(RRDACH)]^+$

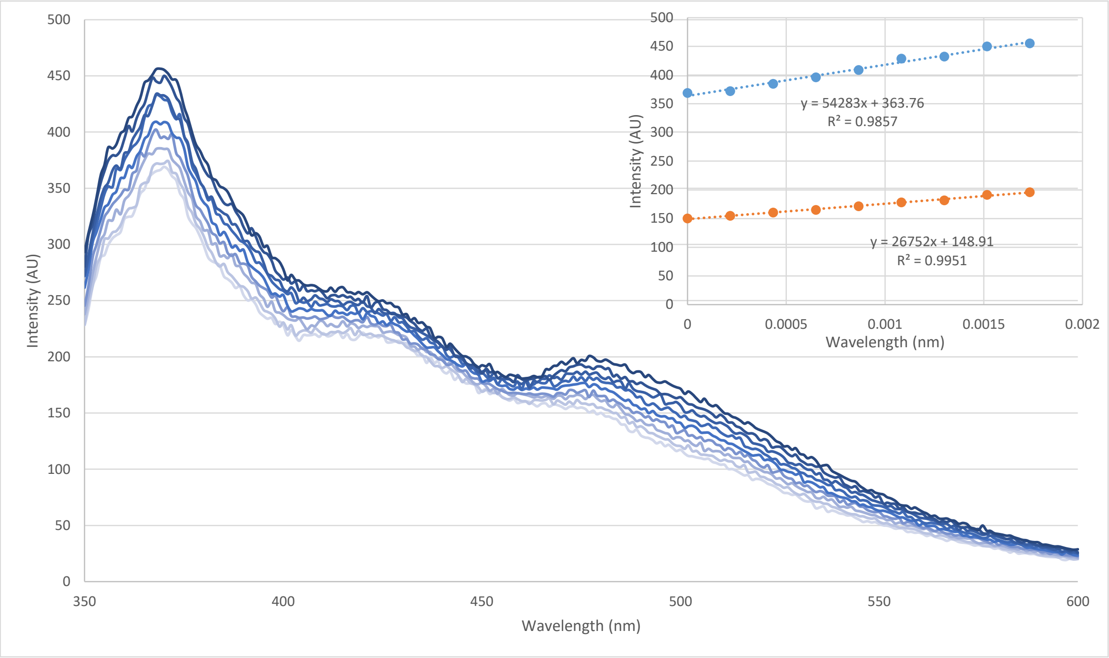

Figure S49: fluorescent intensity of **2b** with increasing concentration of ctDNA in Buffer (blue getting darker as DNA concentration increases). Insert shows graph of fluorescence intensification as concentration of DNA increases at 370 nm (blue) and 480 nm (orange).

$[Pt(DiBequ)(SSDACH)^{]+}$

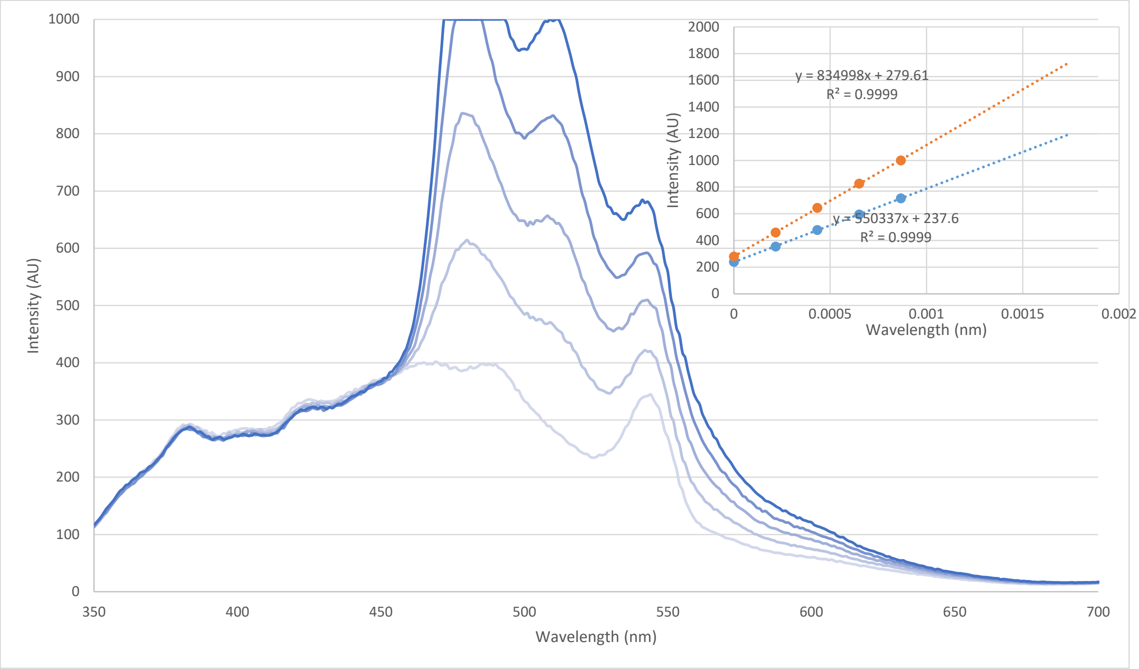

Figure S50: fluorescent intensity of **3a** with increasing concentration of ctDNA in Buffer (blue getting darker as DNA concentration increases). Insert shows graph of fluorescence intensification as concentration of DNA increases at 526 nm (blue) and 511 nm (orange).

[Pt(DiBequ)(RRDACH)]⁺

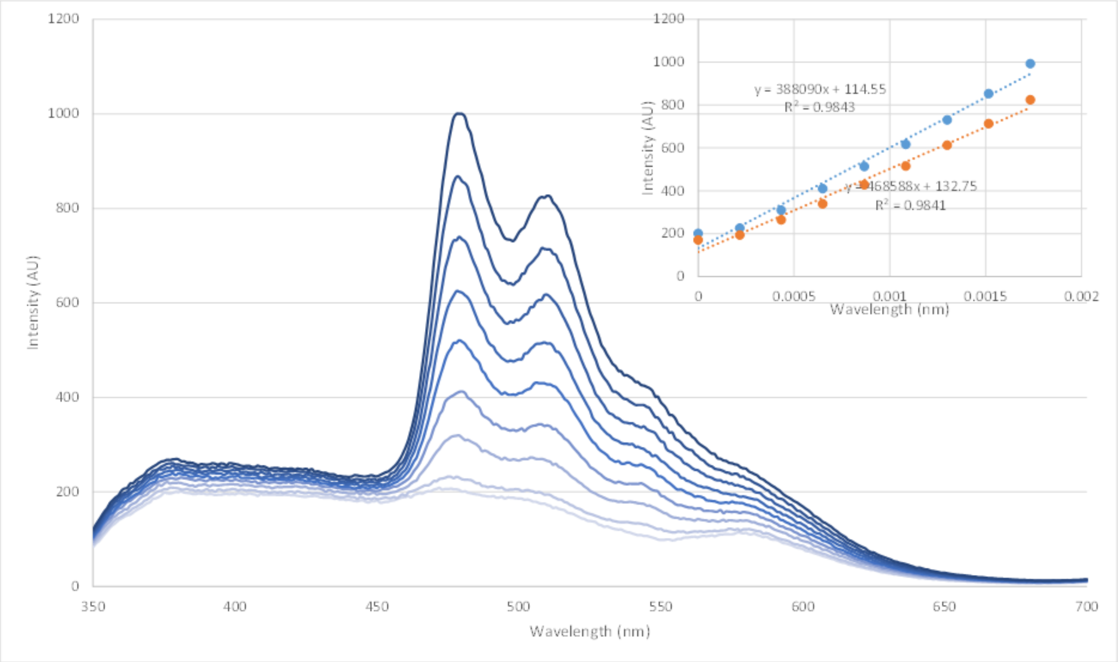

Figure S51: fluorescent intensity of **3b** with increasing concentration of ctDNA in Buffer (blue getting darker as DNA concentration increases). Insert shows graph of fluorescence intensification as concentration of DNA increases at 581 nm (blue) and 511 nm (orange).

Fluorescent Intercalation Displacement Assay

[Pt(Pepy)(SSDACH)]⁺

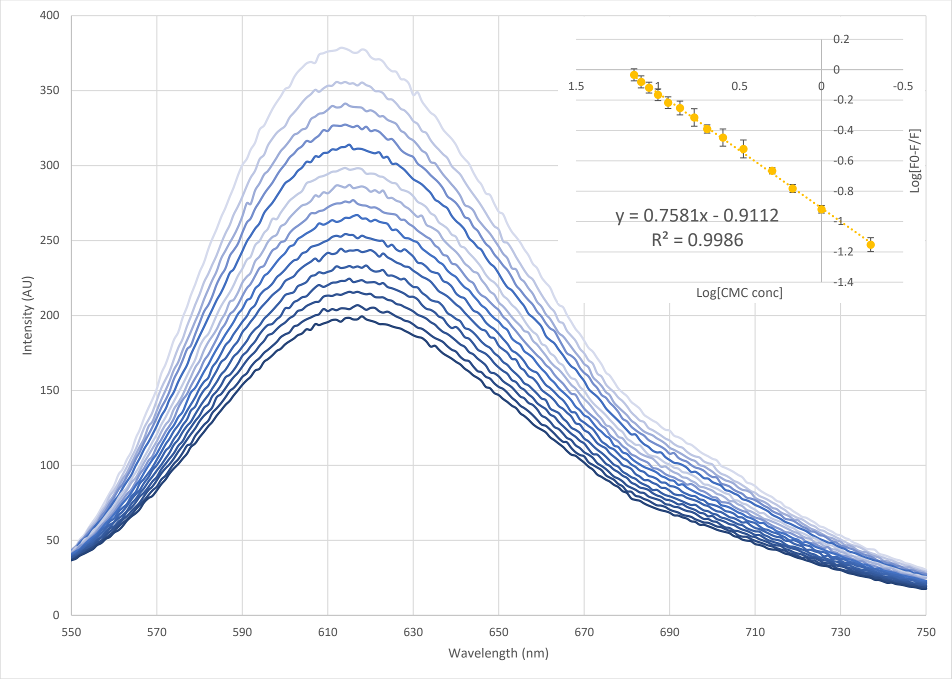

Figure S52: Fluorescent intensity of EtBr saturated ctDNA with increasing concentration of CMC **1a** in Buffer (blue getting darker as CMC concentration increases). Insert shows graph of log of change in fluorescence vs the log of concentration of CMS.

[Pt(Pepy)(RRDACH)]⁺

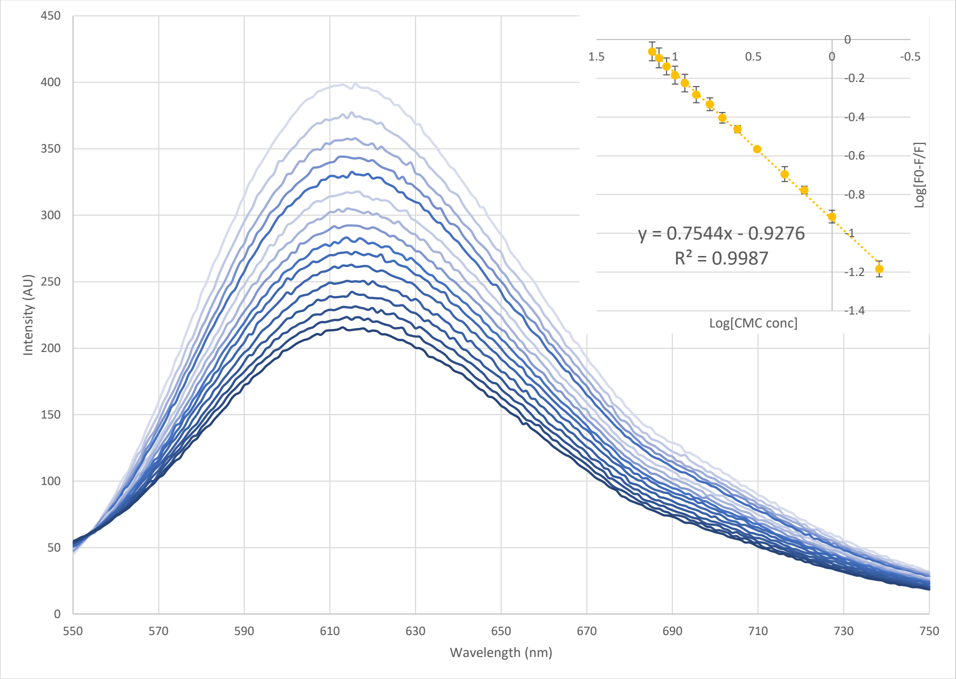

Figure S53: Fluorescent intensity of EtBr saturated ctDNA with increasing concentration of CMC **1b** in Buffer (blue getting darker as CMC concentration increases). Insert shows graph of log of change in fluorescence vs the log of concentration of CMS.

[Pt(Bequ)(SSDACH)]⁺

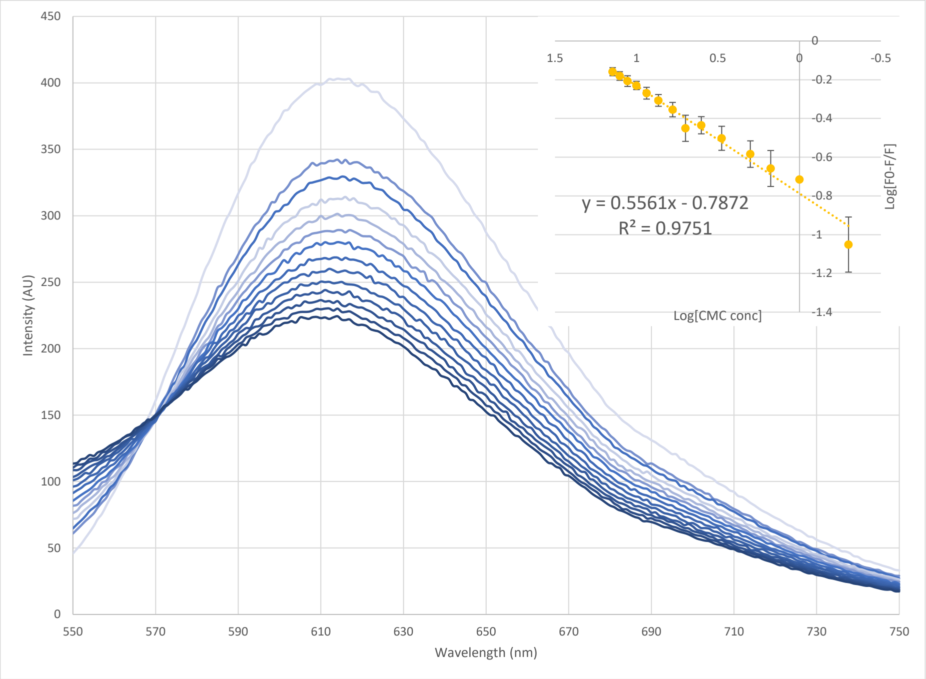

Figure S54: Fluorescent intensity of EtBr saturated ctDNA with increasing concentration of CMC **2a** in Buffer (blue getting darker as CMC concentration increases). Insert shows graph of log of change in fluorescence vs the log of concentration of CMS.

[Pt(Bequ)(RRDACH)]⁺

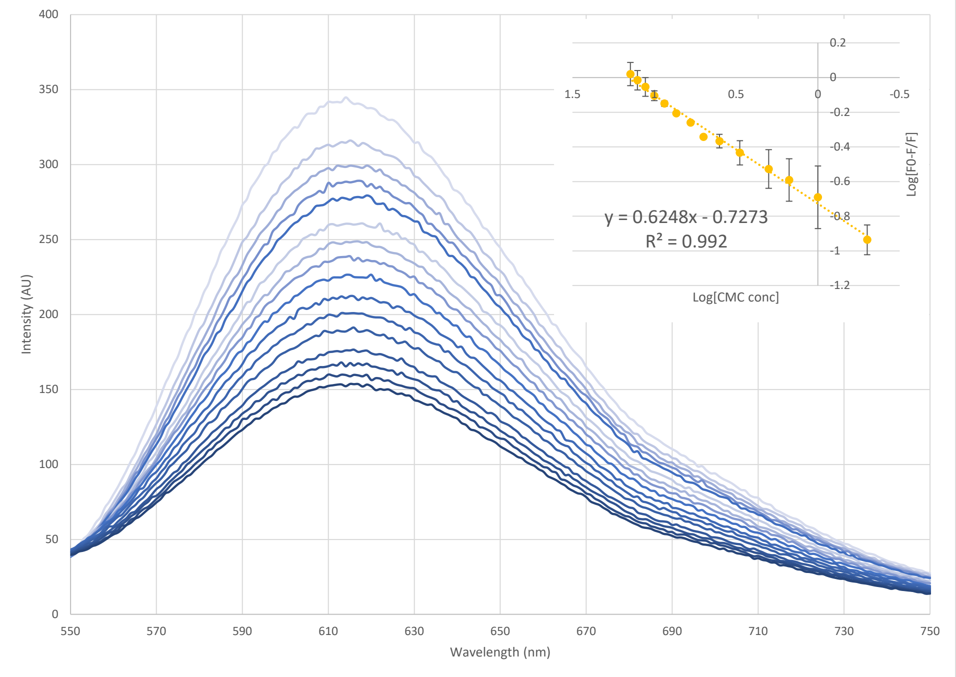

Figure S55: Fluorescent intensity of EtBr saturated ctDNA with increasing concentration of CMC **2b** in Buffer (blue getting darker as CMC concentration increases). Insert shows graph of log of change in fluorescence vs the log of concentration of CMS.

[Pt(DiBequ)(SSDACH)]⁺

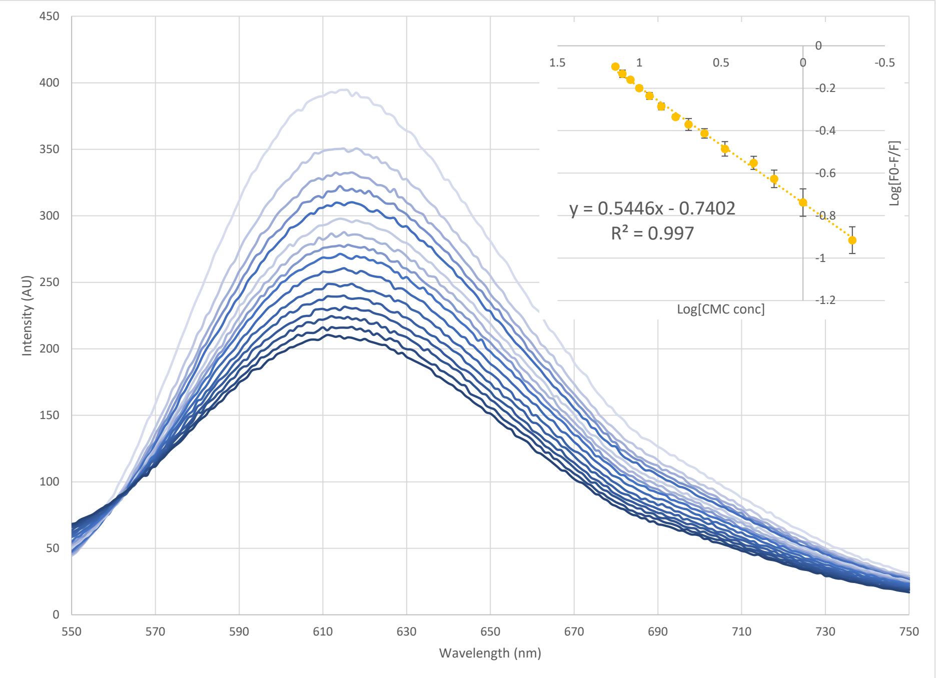

Figure S56: Fluorescent intensity of EtBr saturated ctDNA with increasing concentration of CMC **3a** in Buffer (blue getting darker as CMC concentration increases). Insert shows graph of log of change in fluorescence vs the log of concentration of CMS.

[Pt(DiBequ)(RRDACH)]⁺

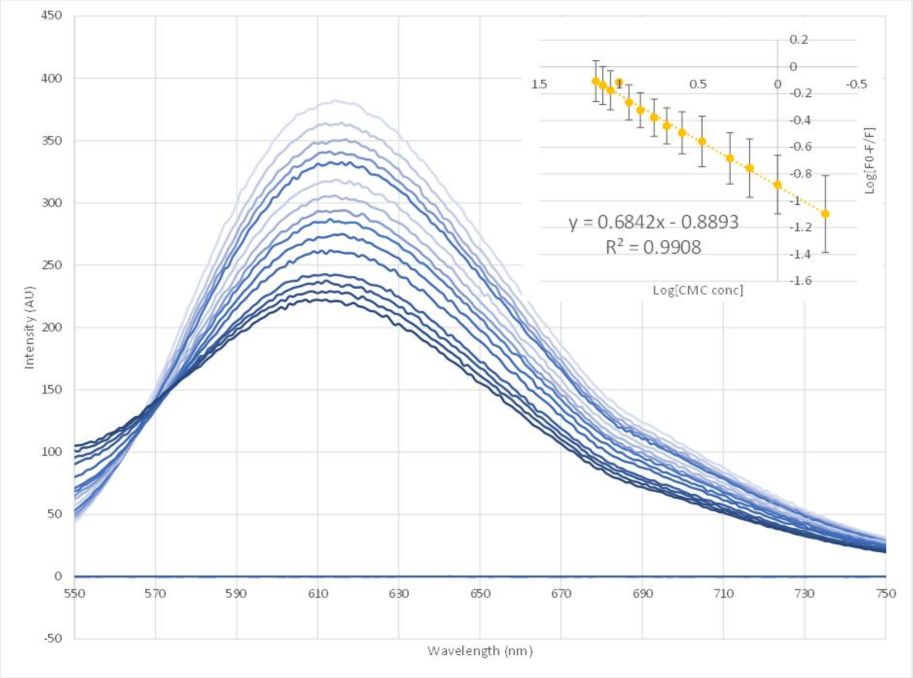

Figure S57: Fluorescent intensity of EtBr saturated ctDNA with increasing concentration of CMC **3b** in Buffer (blue getting darker as CMC concentration increases). Insert shows graph of log of change in fluorescence vs the log of concentration of CMS.

FID vs Inherent fluorescence

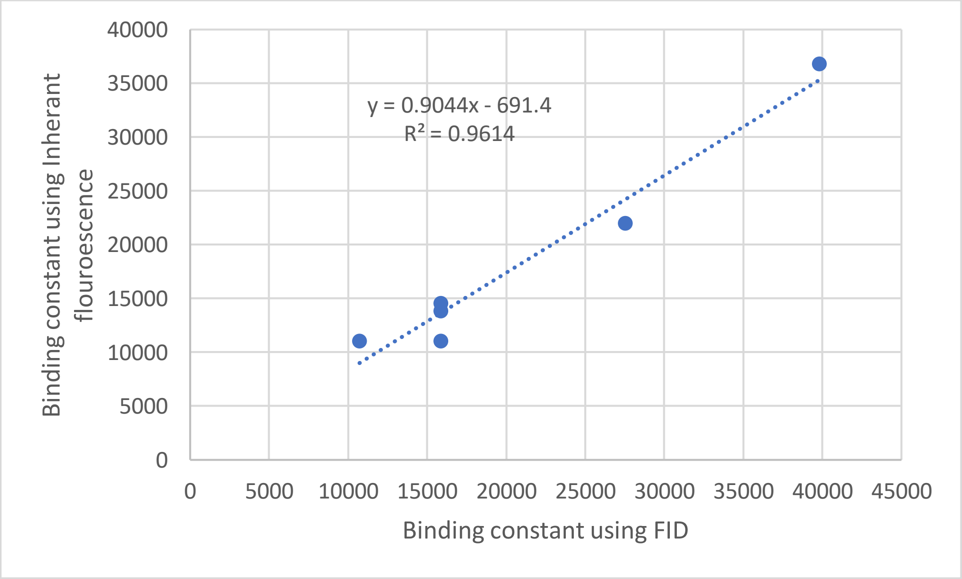

Figure S58: binding constant (Ka) calculated using FID experiments plotted against the binding constant (Ka) calculated using inherent fluorescence experiments.

Cytotoxicity

	GI_{50} Concentration (µM) that inhibits cell growth by 50%					
	**1a**	**1b**	**2a**	**2b**	**3a**	**3b**
**HT29**	13 ± 1.0	41 ± 2.7	16 ± 1.3	30 ± 2.3	16 ± 0.00	16 ± 0.3
**U87**	5.3 ± 0.3	23 ± 2.2	8.6 ± 1.1	4.3 ± 0.3	16 ± 0.6	4.1 ± 0.3
**MCF-7**	8.4 ± 2.0	13 ± 1.7	5.4 ± 1.0	7.7 ± 2.0	15 ± 0.5	4.2 ± 0.3
**A2780**	12 ± 0.7	31 ± 2.9	18 ± 2.3	5.0 ± 0.3	12 ± 0.9	4.5 ± 0.3
**H460**	5.8 ± 0.4	25 ± 2.9	11 ± 1.5	8.1 ± 1.0	16 ± 0.9	8.2 ± 2.9
**A431**	4.2 ± 0.5	29 ± 2.6	12 ± 2.4	3.4 ± 0.3	14 ± 0.6	3.9 ± 0.4
**Du145**	9 ± 2.0	41 ± 0.9	26 ± 2.0	20 ± 2.7	23 ± 2.1	16 ± 1.5
**BE2-C**	34 ± 7.5	38 ± 4.6	23 ± 3.5	48 ± 1.7	9 ± 1.3	33 ± 1.2
**SJ-G2**	11 ± 0.7	20 ± 4.8	13 ± 2.2	10 ± 2.2	17 ± 0.6	10.1 ± 2.6
**MIA**	8 ± 1.6	41 ± 2.0	20 ± 3.0	8.1 ± 1.6	15 ± 0.7	12.5 ± 2.0
**MCF10A**	6.4 ± 0.5	32 ± 2.7	20 ± 1.0	10.3 ± 1.9	16 ± 0.6	8.5 ± 1.4
**ADDP**	5.1 ± 0.3	26 ± 0.6	14 ± 0.3	19 ± 3.0	15 ± 0.00	6.9 ± 0.4
**SI MCF10A** /**MCF**-7	0.76	2.46	3.70	1.33	1.07	2.02

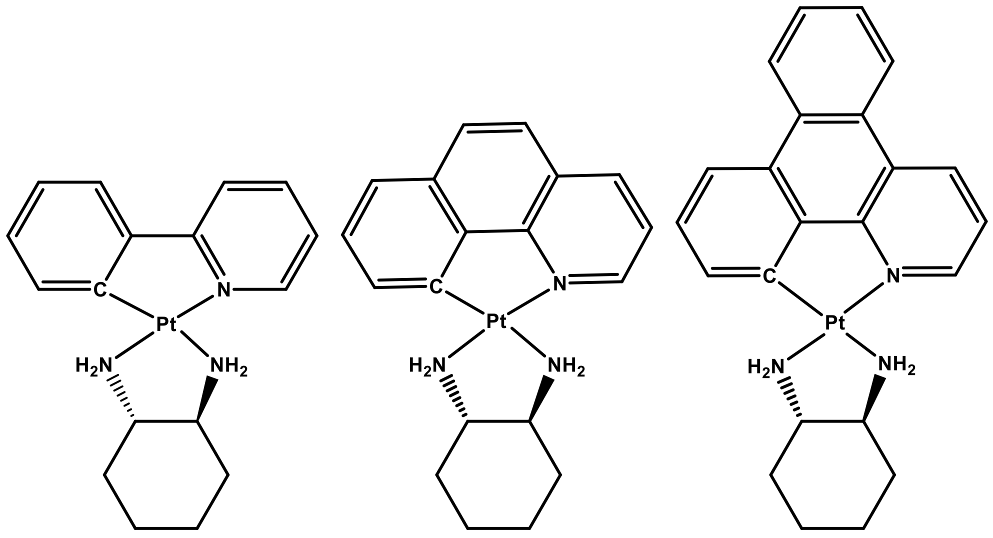

1a **2a** **3a**

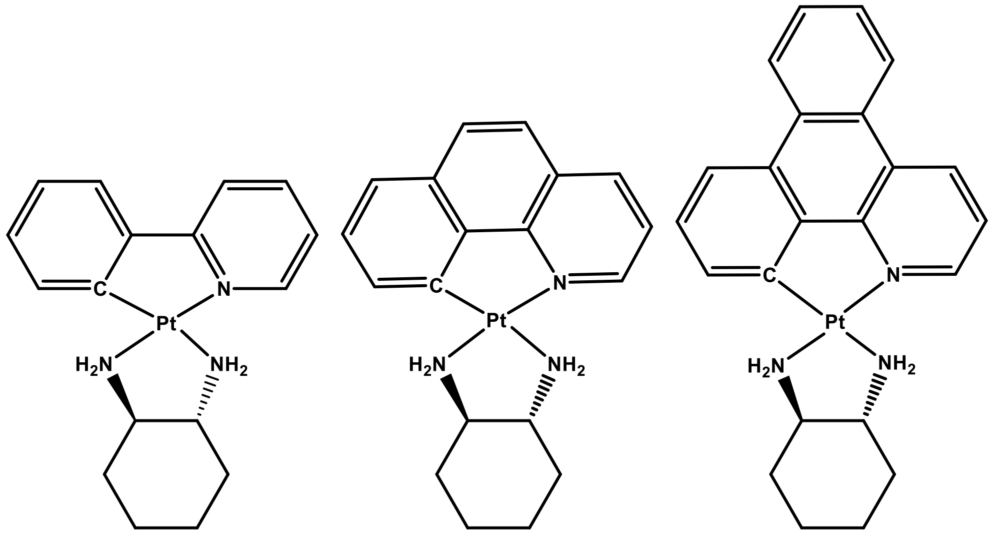

1b **2b** **3b**

Figure S59: structures of each complex, showing stereoisomers.

References

1. Klose, M.H.M.; Theiner, S.; Varbanov, H.P.; Hoefer, D.; Pichler, V.; Galanski, M.; Meier-Menches, S.M.; Keppler, B.K. Development and Validation of Liquid Chromatography-Based Methods to Assess the Lipophilicity of Cytotoxic Platinum(IV) Complexes. *Inorganics* **2018**, *6*, 130, doi:10.3390/inorganics6040130.
2. Reithofer, M.R.; Bytzek, A.K.; Valiahdi, S.M.; Kowol, C.R.; Groessl, M.; Hartinger, C.G.; Jakupec, M.A.; Galanski, M.; Keppler, B.K. Tuning of Lipophilicity and Cytotoxic Potency by Structural Variation of Anticancer Platinum(IV) Complexes. *J. Inorg. Biochem.* **2011**, *105*, 46–51, doi:10.1016/j.jinorgbio.2010.09.006.
3. Valkó, K. Application of High-Performance Liquid Chromatography Based Measurements of Lipophilicity to Model Biological Distribution. *J. Chromatogr. A* **2004**, *1037*, 299–310, doi:10.1016/j.chroma.2003.10.084.
